# A combination of mutational and computational scanning guides the design of an artificial ligand-binding controlled lipase

**DOI:** 10.1038/srep42592

**Published:** 2017-02-20

**Authors:** Marco Kaschner, Oliver Schillinger, Timo Fettweiss, Christina Nutschel, Frank Krause, Alexander Fulton, Birgit Strodel, Andreas Stadler, Karl-Erich Jaeger, Ulrich Krauss

**Affiliations:** 1 Institut für Molekulare Enzymtechnologie, Heinrich-Heine Universität Düsseldorf, Forschungszentrum Jülich GmbH, D-52425 Jülich, Germany; 2 Institute of Complex Systems ICS-6: Structural Biochemistry, Forschungszentrum Jülich GmbH, D-52425 Jülich, Germany; 3 Nanolytics, Gesellschaft für Kolloidanalytik GmbH, Am Mühlenberg 11, 14476 Potsdam, Germany; 4 Jülich Centre for Neutron Science JCNS and Institute for Complex Systems ICS, Forschungszentrum Jülich GmbH, D-52425 Jülich, Germany; 5 Institute of Bio- and Geosciences IBG-1: Biotechnology, Forschungszentrum Jülich GmbH, D-52425 Jülich, Germany

## Abstract

Allostery, i.e. the control of enzyme activity by a small molecule at a location distant from the enzyme’s active site, represents a mechanism essential for sustaining life. The rational design of allostery is a non-trivial task but can be achieved by fusion of a sensory domain, which responds to environmental stimuli with a change in its structure. Hereby, the site of domain fusion is difficult to predict. We here explore the possibility to rationally engineer allostery into the naturally not allosterically regulated *Bacillus subtilis* lipase A, by fusion of the citrate-binding sensor-domain of the CitA sensory-kinase of *Klebsiella pneumoniae*. The site of domain fusion was rationally determined based on whole-protein site-saturation mutagenesis data, complemented by computational evolutionary-coupling analyses. Functional assays, combined with biochemical and biophysical studies suggest a mechanism for control, similar but distinct to the one of the parent CitA protein, with citrate acting as an indirect modulator of Triton-X100 inhibition of the fusion protein. Our study demonstrates that the introduction of ligand-dependent regulatory control by domain fusion is surprisingly facile, suggesting that the catalytic mechanism of some enzymes may be evolutionary optimized in a way that it can easily be perturbed by small conformational changes.

Allosteric regulation represents a general mechanism which is used throughout all kingdoms of life to achieve control of protein activity. In terms of their evolution it appears reasonable to assume that allosteric proteins evolved from non-allosteric ones. Hereby, the evolution of multidomain (sensory) proteins is of particular interest for engineering purposes mimicking natural evolution, as they potentially arose through establishing domain interactions between independently functioning, ancestral proteins^1^,^2^,^3^. Thus, a key to understanding allostery in multidomain sensory proteins is to understand how those proteins gain, lose and rearrange domains. In theory, new functionalities can emerge by at least two mechanisms: i) the interchange of sensor and effector domains between different sensory receptors in a process called domain shuffling^4^ and ii) the recruitment of a sensor domain to an existing non-allosteric protein module^3^. One of the most widespread and versatile sensor domain families, e.g. present in sensory histidine kinases (SHKs)^5^ and other multidomain sensory receptors are Per-Arnt-Sim (PAS) domains^6^. Signal perception by PAS domains is usually determined by covalently or non-covalently bound small molecule ligands^7^. Structurally, PAS domains possess a mixed α/β-fold, where usually five anti-parallel β-strands together with a variable set of α-helices form a tight pocket in which the respective ligand is bound^7^. Known ligands include heme, flavins (flavin mononucleotide, FMN and flavin adenine dinucleotide, FAD), 4-hydroxycinnamic acid (4-HCA), divalent metal cations, C3-C4 carboxylic acids (malonate, malate, succinate), C6 carboxylic acids (citrate)^7^. The environmental stimuli that a given PAS domain can recognize, are equally diverse, ranging from chemical signals such as metabolite concentration (e.g. carboxylic acids)^8^,^9^,^10^, oxygen (heme)^11^,^12^, redox potential (FAD)^13^,^14^ to physical signals such as light (FAD, FMN and 4-HCA)^15^,^16^,^17^. Based on this diversity and the modular nature of PAS-domain containing sensory receptors, efforts have been made recently to engineer allosteric behaviour into naturally non-allosteric proteins by fusion of PAS sensory domains^18^,^19^,^20^. Although successful in several cases^18^,^19^,^20^,^21^,^22^,^23^,^24^,^25^, the rational engineering of allostery into an existing non-allosteric protein still represents a challenging endeavour. Several strategies have been brought forward all relying on the above described two evolutionary mechanisms, i.e. domain swapping to reprogram allosteric control altering the sensory input of the system^24^,^25^, insertion^18^,^23^ or terminal fusion^19^,^20^ of a sensory domain. Often, the screening of several fusion constructs^20^,^22^,^23^,^24^ and/or circular permutation and several rounds of directed evolution^26^ were necessary to obtain an efficient switch. Thus, the general question arises, which of the already explored strategies represents the best for a given target protein, and, more importantly, is it possible to rationally predict the best strategy (N-terminal fusion, C-terminal fusion or insertion) based on i.e. bioinformatics analyses or already available mutational data for a given target protein? To this end, several bioinformatic methods have been developed that infer the evolutionary (statistical) coupling between residue pairs in a given protein family sequence alignment^27^,^28^. It is reasoned, that this co-evolutionary information captures the statistical signature of functional constraints arising from conserved communication between positions and thus enable the identification of chains of residues facilitating the flow of information necessary for allosteric communication^18^,^27^,^28^,^29^.

In the present contribution, we explore the possibility of rationally engineering allosteric control into the naturally not allosterically regulated lipase A of the Gram-positive bacterium *Bacillus subtilis* (BsLA)^30^ by fusion of the citrate-binding CitAP PAS domain of the periplasmic CitA citrate-sensor of *Klebsiella pneumonia*^9^, hereby mimicking evolutionary processes that could lead to the emergence of new multidomain proteins. The site of domain fusion (N-terminal, C-terminal or insertion) was rationally determined based on a whole-protein site-saturation mutagenesis dataset of BsLA, backed by computational evolutionary coupling analysis. Functional assays, complemented by a set of biochemical and biophysical studies, suggest a mechanism for control of the artificial citrate-binding lipase, similar but distinct to the one suggested to be realized in the parent sensor-domain containing CitA SHK. Our study demonstrates that the generation of ligand-binding dependent control of an enzyme by sensory domain fusion can easily be achieved in a simple “plug and play” manner.

## Results

### Computational predictions and site-saturation scanning mutagenesis data identify a network of functionally and evolutionary coupled residues at the N-terminus of BsLA

**Figure 1:**
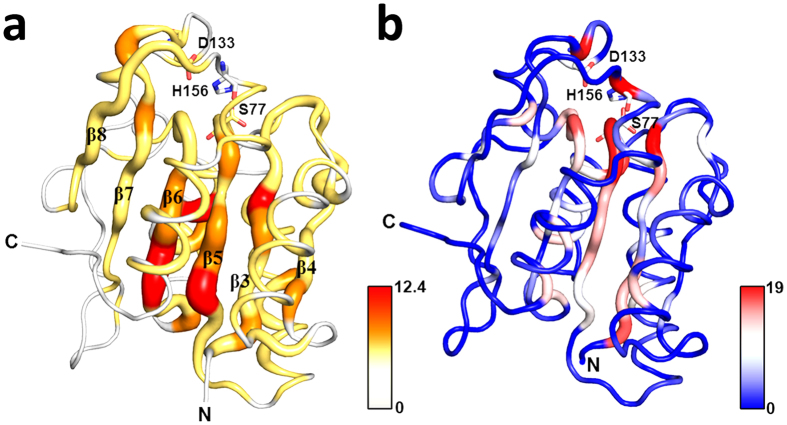
Comparison of evolutionary-coupling analyses **(a)** and site-saturation scanning mutagenesis data **(b)** mapped onto the X-ray structure of BsLA. Evolutionary coupled residues were inferred from a multiple sequence alignment using the EVcoupling webserver (www.evfold.org). The obtained evolutionary constraints (EC) values were mapped onto the X-ray structure of BsLA (PDB Entry: 1I6W)^30^. The magnitude of the obtained EC scores is color-coded (low values in yellow; high values in red). Additionally, EC values are encoded by sausage thickness representing the magnitude of the EC score. For orientation, the central β-scaffold (β3- β8) of BsLA is labelled according to topological order^30^. The number of inactive BsLA variants per residue was obtained from a complete site-saturation mutagenesis dataset (**b**) and mapped onto the BsLA X-ray structure. The number of inactive variants is encoded by color (blue: low values; red: high values) and sausage thickness. The N- and C-termini of BsLA are indicated. The residues of the catalytic triad, Ser77, Asp133 and His156 are shown as sticks with oxygen in red, carbon in grey and nitrogen atoms in blue. The color-bars next to the respective figure represent the plotted scale of EC values and the number of inactive BsLA variants per mutated site.

BsLA is a monomeric α/β-hydrolase that hydrolyses glycerol-esters with medium chain length (C8) as well as *sn-1* and *sn-3* glycerol esters with long fatty acid chains to the corresponding alcohols^30^. It is one of the smallest known lipases, that, in contrast to other lipases, lacks a lid-domain structure and hence does not show interfacial activation^30^. No allosteric effects have so far been described for BsLA. In order to infer chains of evolutionary coupled residues and hence to identify the best site for sensor domain fusion, we computationally inferred the evolutionary coupling between residues in BsLA by using the EVcoupling webserver (www.evfold.org)^31^,^32^. In order to obtain reliable evolutionary constraints (EC) values, we constructed a large hydrolase core alignment with the BsLA sequence as query for alignment generation using the tools available as part of the EVcoupling webserver. In an unrestrained run, an alignment containing 149.524 sequences was generated (E-value cutoff 10E-3) which was subsequently used to infer EC scores for every residue in the conserved BsLA core. The resulting EC values were mapped onto the BsLA X-ray structure (Fig. 1a, see also Supplementary Figure 1). Evolutionary coupled residues are color-coded from grey (low EC values) to red (high EC values). A network of evolutionary coupled residues appears to be centred around the anti-parallel β-scaffold of BsLA, with the highest values obtained for residues on β3, β5, β6. To experimentally validate those findings we used a set of data obtained by complete site-saturation mutagenesis of BsLA^33^,^34^ and parsed this data for residues whose substitution led to severe loss of function. From this data, the number of inactive variants per residue was determined (Supplementary Figure 2) and the respective values were mapped on the X-ray structure of BsLA (Fig. 1b). Interestingly, very similar to the data obtained from evolutionary-coupling analyses, most “mutationally-sensitive” residues, i.e. those where mutations led in many cases to loss of enzyme activity, are found within the β-scaffold of BsLA, namely on strands β3, β5, β6. In particular, the first N-terminal 11 amino acids including the β3 strand (residues 6 to 9) appear especially sensitive to mutation. Importantly, a similar network of functionally important residues seems to be absent at the C-terminus or within loop regions of BsLA.

### Design of the fusion protein

**Figure 2:**
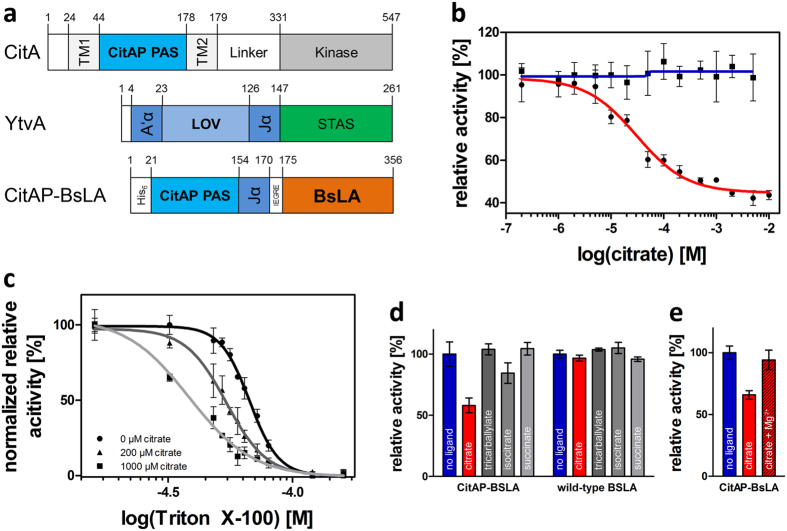
(**a**)Schematic representation of the multidomain architecture of the sensory histidine kinase CitA of *Klebsiella pneumoniae*, the blue-light photoreceptor YtvA of *Bacillus subtilis* and the here constructed artificial ligand-binding controlled lipase CitAP-BsLA. The numbers above the boxes denote amino acid numbers at domain boundaries of the respective full-length proteins. Abbreviations: TM1, TM2: transmembrane helices, CitAP PAS: periplasmic citrate-binding sensory domain of CitA, A’α: N-terminal N-cap α-helix of YtvA, LOV: blue-light sensing light oxygen voltage domain of YtvA, Jα: α-helical linker connecting LOV and STAS domains of YtvA, STAS: sulfate-transporter anti-sigma factor antagonist domain, His_6_: Hexa-histidine tag, IEGRE: protease Factor Xa cleavage site, BsLA: *B. subtilis* Lipase A. Domain boundaries of CitA according to Kaspar *et al*.^37^. **(b)** The lipolytic activity of CitAP-BsLA (red-line, black circles) and wild type BsLA (blue-line, black squares) were determined in the presence of increasing concentrations of sodium citrate. The experimental data was fitted using a four parameter logistic dose-response model (red line). **(c)** The activity change in the presence of increasing concentrations of Triton X-100 was determined at 0 (black line, circles), 200 μM (dark grey line, triangles) and 1000 μM sodium citrate (light grey line, squares). **(d)** Sodium citrate, as well as the respective citrate analogues, were added to the assay in a final concentration of 1 mM and the lipolytic activity of CitAP-BsLA and BsLA was determined relative to the activity without ligand **(e)** The presence of Mg^2+^ ions, which are known to scavenge citrate^37^, abolishes the functional response of CitAP-BsLA. 10 mM MgCl_2_ was added to the assay containing CitAP-BsLA and 1 mM sodium citrate. Lipolytic activity was measured using *p*-nitrophenylbutyrate as substrate. Error bars depict the standard deviation of the mean derived from three independent measurements.

Based on the above described analyses, a potential allosteric communication pathway was predicted extending from the BsLA N-terminus *via* the first β-strand to the enzyme active site (Fig. 1). Thus, in order to gain control over BsLA function we fused the citrate-binding PAS domain CitAP of the CitA SHK of *Klebsiella pneumoniae*^9^ N-terminally to BsLA as a putative “effector” module. Hereby, the CitAP PAS domain (residues 44 to 178 of full-length CitA) and full-length BsLA were linked *via* the Jα linker (residues 126 to 147) of the *B. subtilis* YtvA photoreceptor^35^, resulting in a tripartite fusion protein (Fig. 2a). In wild-type CitA, a transmembrane helix (TM2) connects the periplasmic CitAP PAS sensor domain and the cytosolic histidine kinase (HK) effector domain (Fig. 2a). We decided to replace this TM2 helix (residues 179 to 199) of wild-type CitA by the YtvA Jα linker, to allow for soluble expression in *E. coli*. As suggested for full-length CitA, we reasoned, that in the here designed, potentially ligand-binding controlled lipase, the conformational change induced by ligand binding in the CitAP PAS domain could be transmitted *via* the Jα linker to affect BsLA activity.

### Lipase activity of CitAP-BsLA depends on citrate

The gene-fusion coding for CitAP-BsLA was expressed in *E. coli* as a hexa-histidine (His6)-tagged fusion protein and purified to homogeneity by immobilized metal affinity chromatography and preparative size exclusion chromatography. A specific activity of 509 ± 5 U/mg was determined for purified CitAP-BsLA, while purified wild-type BsLA showed an activity of 181 ± 3 U/mg with *p*-nitrophenylbutyrate as a model substrate. This suggests that fusion of CitAP to BsLA had no negative influence on the lipolytic activity of BsLA. On the contrary, the specific activities of CitAP-BsLA exceeded those of the isolated wild type BsLA. This observation might be related to the fact that fusion of CitAP to BsLA results in an increased solubility of the protein. While BsLA starts to aggregate at pH 10 at concentrations higher than 1 mg/ml, CitAP-BsLA can easily be concentrated to 5–10 mg/ml (data not shown). The effect is even more pronounced at neutral pH values, i.e. under assay conditions. This might result in higher stability of the fusion protein under assay conditions and thus could account for the increased apparent specific activity.

To address citrate sensitivity of CitAP-BsLA, we performed lipase assays in the presence of different concentrations of sodium citrate. Figure 2b shows the dose response curve recorded for the citrate-dependence of CitAP-BsLA lipase activity, displaying a clear sigmoidal response, characteristic for specific binding interactions and ligand-dependent functional regulation (Fig. 2b; red line). In contrast, isolated wild-type BsLA, without attached sensor domain, did not show any response toward citrate in the tested concentration range (Fig. 2b; blue line). From the fit of experimental data, an apparent K_D_ of 32 ± 8 μM and a Hill coefficient (*n*_*H*_) of 0.94 ± 0.11 can be derived. During setup of the lipase assay for CitAP-BsLA, we realized, that the detergent Triton-X100 (TX100), which is added to the assay to solubilize the hardly water-soluble lipase substrate, apparently influences the magnitude of the functional citrate dependent response of CitAP-BsLA. We therefore performed an experiment where we kept the citrate concentration constant but varied the TX100 concentration in the assay (Fig. 2c). Please note, that the maximally employed TX100 concentration (160 μM) is well below the critical micelle concentration (CMC) of the detergent (0.22 mM)^36^. In this way, we are able to derive dose response curves for the TX100-dependent response of CitAP-BsLA at three different sodium citrate concentrations (Fig. 2c). The dose response curves display sigmoidal character, indicative of specific binding of TX100 to the protein. At different citrate concentrations, different apparent K_D_ and *n*_*H*_ values are obtained. At a concentration of 1 mM citrate, an apparent K_D_ of 38 ± 1 μM can be derived, with a Hill coefficient of 3.64 ± 0.42. In the absence of citrate the K_D_ is increased to 67 ± 1 μM (*n*_*H*_ = 7.53 ± 0.42), revealing an increased inhibitory potential for TX100 in the presence of citrate. In order to further analyze the role of the detergent TX100 on the citrate dependent activity response of CitAP-BsLA and wild type BsLA, we determined the functional response, i.e. the lipolytic activity in the presence and absence of 1 mM citrate, at different TX100 concentrations (Supplementary Figure 10). While the measurement conducted using wild type BsLA does not show a clear TX100 dependency and a relatively large associated error, the measurement for CitAP-BsLA reveals a maximal activity response at approx. 50 μM TX100. In the absence of the detergent no functional response of CitAP-BsLA is observed. In light of those findings, the observed citrate-dependent reduction of CitAP-BsLA lipase activity has to be interpreted as a citrate-dependent modulation of TX100 inhibition of CitAP-BsLA.

### CitAP-BsLA fusion and isolated CitA display similar ligand-binding characteristics

The specifity of CitAP-BsLA was further probed by using different citrate analogues. The isolated CitAP sensor domain was reported to be highly specific for citrate^37^. We therefore used isocitrate, succinate and tricarballylate as potential ligands and analysed the functional response of CitAP-BsLA. As expected for a highly specific citrate-sensor, CitAP-BsLA did not respond to any of the tested analogues (Fig. 2d). Similarly, the isolated BsLA protein did not show any change in activity due to presence of citrate analogs (Fig. 2d). Moreover, it was reported that Mg^2+^ ions can form a stable complex with citrate^37^. Therefore, the addition of MgCl_2_ to the assay solution is expected to inhibit the citrate-dependent functional response of CitAP-BsLA by interfering with citrate-binding. As expected, addition of 10 mM MgCl_2_ to the assay solution containing 1 mM citrate completely abolished the functional response of CitAP-BsLA (Fig. 2e). Please note that, all experiments using citrate analogues and MgCl_2_ were performed in the presence of TX100, which indicates that the detergent does not influence the ligand-binding properties of the CitAP domain in CitAP-BsLA, i.e compared to the isolated CitAP domain.

### Global citrate-induced structural changes in CitAP-BsLA probed by fluorescence spectroscopy

**Figure 3:**
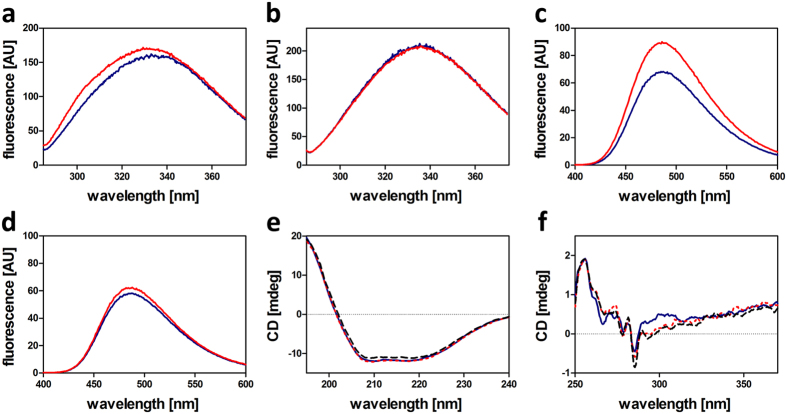
Fluorescence of aromatic amino acids of CitAP-BsLA **(a)** and wild-type BsLA **(b)**. Protein samples, diluted to 3 μM in 10 mM glycin buffer pH 10 supplemented with 10 mM NaCl, were excited at 278 nm. **(c)** Fluorescence emission spectra of 4,4′-dianilino-1,1′-binaphthyl-5,5′-disulfonic acid (bis-ANS) of samples containing CitAP-BsLA or wild-type BsLA **(d)**. 6 μM of bis-ANS was added to protein samples (3 μM) in 10 mM glycin buffer pH 10 supplemented with 10 mM NaCl. Bis-ANS was excited at 385 nm. All fluorescence emission spectra were recorded in the presence (red line) and absence (blue line) of 1 mM sodium citrate. Far-UV **(e)** and near-UV **(f)** circular dichroism (CD) spectra of CitAP-BsLA in 10 mM glycine buffer pH 10 supplemented with 10 mM NaCl (blue solid line), after addition of 1 mM sodium citrate (red dashed line) and in the presence of 1 mM sodium citrate and 50 μM Triton X-100 (black dashed line). All spectra were recorded at 25 °C.

In order to assess global structural changes in CitAP-BsLA induced by citrate binding we initially monitored the fluorescence of the aromatic amino acid residues of CitAP-BsLA and wild-type BsLA. Excitation of tryptophan (Trp) residues of CitAP-BsLA and wild-type BsLA at 295 nm did not reveal any spectral changes due to the presence of citrate (Supplementary Figure 3). In contrast, excitation at 278 nm, thus exciting both tyrosine (Tyr) and Trp residues, resulted in distinctly different emission spectra for samples with and without 1 mM citrate. In the presence of 1 mM citrate, an increased emission (Fig. 3a) with a maximum at around 303 nm is observed for CitAP-BsLA (maximum derived from the resulting difference spectrum, (data not shown). This small, but reproducible, increase in fluorescence emission could be attributed to an increased emission from Tyr residues and thus to reduced Förster-Resonance-Energy Transfer (FRET) between Tyr and Trp residues in the protein. In contrast, no citrate-dependent change in Tyr-Trp FRET was observed for the isolated BsLA protein (Fig. 3b). To further probe global structural changes in CitAP-BsLA we employed the fluorescent dye 4,4′-dianilino-1,1′-binaphthyl-5,5′-disulfonic acid (bis-ANS)^38^,^39^, which binds to hydrophobic surface patches of proteins^40^. Upon dye binding, an increased fluorescence emission as well as a blue-shift of the emission maximum, compared to the free dye, can be observed. Bis-ANS emission was markedly increased for CitAP-BsLA samples containing 1 mM citrate (Fig. 3c), suggesting that upon citrate binding additional hydrophobic surface patches become exposed.

In contrast, only a negligible citrate-dependent change in bis-ANS fluorescence was observed for a sample of the isolated wild-type BsLA protein (Fig. 3d).

### Far- and near-UV circular dichroism (CD) spectroscopy hint at citrate-induced conformational changes

Far-UV CD spectroscopy was used to analyse CitAP-BsLA for potential secondary structural changes associated with citrate-binding. Additionally, due to the observed effect of TX100 on BsLA activity a CitAP-BsLA sample containing the detergent was included (Fig. 3e). Far-UV CD spectroscopy suggested that CitAP-BsLA is well folded in solution but does not reveal any significant secondary structural changes due to presence of sodium citrate or TX100. This notion is further corroborated by deconvolution of the corresponding CD spectra (Supplementary Table 3) and a comparison to the theoretical secondary structure composition of the fusion protein derived from the X-ray structures of the components (Supplementary Table 4). This further suggests that TX100 does not influence the proper folding of CitAP-BsLA. In contrast, near-UV CD spectra revealed citrate-dependent tertiary structural changes independent of the presence of TX100 (Fig. 3f). In the presence of citrate, we observed a decrease in ellipticity at around 265 nm and increased values at 285 nm as well as in the region between 290 nm and 310 nm. While the changes seen at around 285 nm may be attributed to a rearrangement of Tyr side chains which are distributed throughout the whole fusion protein (15 residues), the most pronounced citrate-dependent spectral changes are observed in the 290 nm–310 nm region corresponding to the absorption band of Trp residues. Since Trp residues are only found within the BsLA domain (W31 and W42 of BsLA) of the fusion protein, those spectral changes must be interpreted as a tertiary structural change in the BsLA part of the construct.

### Citrate-dependent quaternary structural changes studied by small angle X-ray scattering (SAXS) and analytical ultra-centrifugation (AUC)

Many, though not all, bacterial SHKs are functionally active as dimers. In those cases, signal relay was suggested to occur *via* a rotation/piston/torque-like movements^41^,^42^,^43^ initiated in the sensor domains which are transduced through rigid coiled-coils in case of soluble SHKs, or transmembrane (TM) helices in case of membrane bound SHKs^44^,^45^,^46^. Given that CitAP is reported to be a dimer^9^,^46^, while BsLA appears to be monomeric, the question arises whether the fusion protein CitAP-BsLA is a monomer or dimer in solution. We therefore initially used AUC to determine the oligomerization state of CitAP-BsLA in solution with or without 1 mM citrate for samples of low concentration (0.5 mg/ml) and subsequently employed SAXS to cover a broader concentration range (0.5–5 mg/ml) to address the possibility of concentration-dependent oligomerization and obtain a low-resolution structural model of the fusion protein. AUC and SAXS data for CitAP-BsLA are summarized in Supplementary Figure 4 and Supplementary Figure 5, respectively. At low concentrations, both AUC and SAXS reveal the presence of monomeric and dimeric species of CitAP-BsLA (Table 1). Moreover, both methods provide an identical estimation of the relative monomer:dimer distribution with the monomer (SAXS: 73%, AUC: 73%) representing the predominant species in the absence of citrate. At low concentrations, this equilibrium is slightly influenced by the presence of citrate, shifting the equilibrium further toward the monomer (SAXS: 86%, AUC: 85%) (Table 1). Additionally, a small but significant citrate-dependent increase in the sedimentation coefficient of the CitAP-BsLA monomer/dimer is observed in AUC experiments (Table 2). Moreover, a citrate-dependent change in the AUC determined frictional ratio f/f_0_ can also be seen in the radius of gyration (R_g_) and the maximal elongation of the molecule (D_max_), derived from SAXS experiments (Table 2). Thereby, the frictional ratio reflects both the shape and hydration of the protein molecule and can be considered as an approximate measure of the molecules’ globularity. Here, a smaller frictional ratio (f/f0) is observed in the presence of citrate indicative of a more globular conformation and/or lower hydration. This observation is corroborated by the SAXS data, where smaller R_g_ and D_max_ values are found in the presence of citrate for the monomer. Please note that we cannot rule out that the change in R_g_ and D_max_ observed by SAXS is caused by the altered monomer:dimer ratio between citate-free and citate-bound protein samples (*vide supra*).

### Dimerization of CitAP-BsLA depends on protein concentration

**Figure 4:**
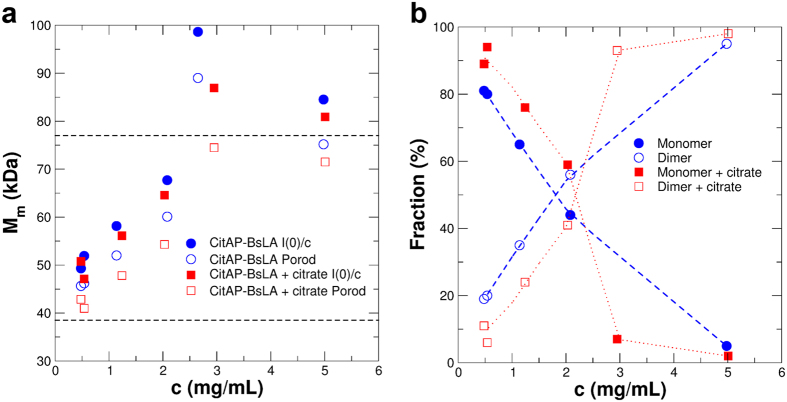
(**a**)Average molecular mass of the scattering particle determined from the concentration normalized forward scattering I(0)/c or from the Porod volume. **(b)** Monomer and dimer fraction of CitAP-BsLA as a function of the protein concentration. Values were determined using the *M*_*m*_ determined from the Porod volume. The respective values were obtained by analysing SAXS data recorded for CitAP-BsLA in the absence (blue) and presence (red) of 1 mM sodium citrate.

SAXS measurements provide direct information about the oligomerization state of a protein. Here, the average molecular mass of the scattering particle was calculated (Fig. 4a) from the forward scattering *I*(0)/*c* normalized by the protein concentration *c*, which is directly proportional to the molecular mass *M*_*m*_ of the scattering particle, and by the Porod volume multiplied with the appropriate protein density^47^. By comparison of scattering curves for CitAP-BsLA at different protein concentrations, in both the absence and the presence of 1 mM citrate, a concentration dependent monomer:dimer equilibrium was observed (Fig. 4b). At all protein concentrations, this equilibrium was shifted by the presence of citrate, resulting in a reduction of the dimer content (Fig. 4b). At concentrations of about 5 mg/ml more than 90% of CitAP-BsLA was present as a dimer. Additionally, from Fig. 4b, a dissociation constant of approximately 1.8 to 2.2 mg/ml (24–29 μM) for the dimer can be estimated, indicating that dimer association is rather weak. Probably, this is a direct consequence of fusing monomeric BsLA to dimeric CitAP thus altering the dimer forming capacity of CitAP by presenting non-evolved protein-protein interaction via the BsLA part of the fusion protein. Given the rather high dissociation constant of the dimer, it seems reasonable to assume that under assay conditions (at 1 μM protein concentration)) CitAP-BsLA is present as a monomer. This implies that the citrate-induced structural changes in monomeric CitAP-BsLA are sufficient to induce the observed functional response.

### Computational modelling and SAXS envelope reconstructions

**Figure 5:**
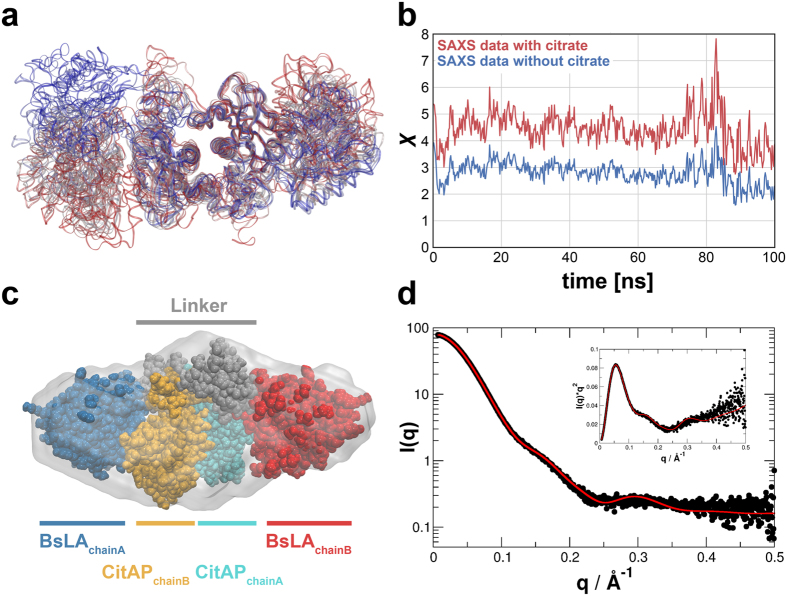
(**a**) Structural changes during the molecular dynamics (MD) simulations of the dimeric M_low-free_ CitAP-BsLA model. The proteins are shown as ribbons and the colors represent structures at different times, changing from red at t = 0 ns to blue at t = 100 ns. (**b**) Time evolution of χ during the MD simulations of the M_low-free_ model. Deviation between the model and experimental data measured at 5 mg/ml (blue) and without citrate (red) are shown. (**c**) Final model of the CitAP-BsLA dimer complex superimposed onto the SAXS derived low-resolution envelope obtained from SAXS data at high protein concentration in the presence of citrate. (**d**) SAXS scattering curve recorded for CitAP-BsLA at a protein concentration of 5 mg/ml in the presence of citrate (black dots) and the CRYSOL-fitted theoretical scattering curve of the final CitAP-BsLA dimer model (red line).

In order to gain more insight into the structural arrangements of CitAP and BsLA in the monomer as well as the assembly of the CitAP-BsLA dimer, we reconstructed low-resolution bead models from SAXS data, further investigated the resulting models using molecular dynamics (MD) simulations, and compared the final MD-derived models to the experimental data obtained in SAXS experiments of CitAP-BsLA with and without citrate. Four different starting models of the dimeric CitAP-BsLA complex were obtained using different strategies. Details about model generation can be found in the Materials and Methods section and the Supplementary Materials. The models differed with regard to the manner of generation and the conformation of the CitAP-PAS domain, being either in the citrate-bound (closed) (models: M_low-cit_, M_high-cit_) or citrate-free (open) state (models M_low-free_, M_high-free_) (Supplementary Table 2). In order to improve the initial models, a 100 ns MD simulation was performed for each dimeric assembly (Supplementary Figure 7). To evaluate the quality of the resulting models sampled during the MD runs, a theoretical scattering curve was calculated for every 200 ps snapshot of each trajectory and fitted against the experimental data (with and without citrate) (Supplementary Figure 8). Hereby, only the MD simulation of M_low-free_ yielded acceptable χ values, as a measure of the goodness of the fit between the experimental data and the theoretical model. Thus, only the data of the M_low-free_ simulation is summarized in Fig. 5. The respective data for all models is given in the Supplementary Materials (Supplementary Figures 7 and 8). During the MD simulation, M_low-free_ (Fig. 5a) and most of the other models (Supplementary Figure 7) underwent significant structural rearrangements. Figure 5b depicts the evolution of χ during the M_low-free_ MD simulation. The corresponding data for the alternative models is shown in Supplementary Figure 8. Hereby, the MD-derived models were compared against the experimental SAXS data measured at protein concentrations at which CitAP-BsLA is predominately dimeric (5 mg/ml), both in the presence (blue) and absence of citrate (red) (Fig. 5b and Supplementary Figure 8). For both cases, the data shows the same overall trend and appears to be scaled by a constant factor, indicating a better experimental accuracy of the scattering curves obtained in absence of citrate and hence a larger χ value. The similar trends in both datasets are likely due to highly similar structures of the fusion protein dimers with and without citrate. After a structural rearrangement at around 80 ns, the χ value reaches a minimum of 1.50 when compared to the experimental scattering data at in the presence of citrate. Thus, this model appears closest to the physical structure of the fusion protein. This model was further optimized by constructing symmetric dimers by superimposing chain A onto the Cα atoms of chain B and *vice versa*. This yielded a structure with a χ value of 1.3 when chain A is superimposed onto chain B. A subsequent energy minimization of this model further improved χ to 1.16. The resulting structure represents the best model in terms of χ and is thus taken as the final model (Fig. 5c). Models from the last frame of each MD simulation are shown in Supplementary Figure 9. For comparison, the MD-derived final model was fitted to a low-resolution SAXS envelope obtained from *ab initio* bead-modelling (Fig. 5c). For the final model, the maximal elongation (D_max_) and the radius of gyration (R_g_) were calculated and compared to the corresponding experimental values. Both values (D_max_ = 12.2 nm; R_g_ = 3.23 nm) are in good agreement with the corresponding experimentally derived values (D_max_ = 11.6 nm; R_g_ = 3.44 nm; see Table 2). As depicted in Fig. 5d, the corresponding theoretical scattering curve agrees nicely with the experimental one.

## Discussion

The computational prediction of allosteric communication pathways in signalling proteins represents an important line of investigation in both basic science and applied pharmaceutical research either enabling or facilitating the design of inhibitors for a given pharmaceutical target. Likewise, the rational design of allosteric communication, so far successful in a few cases only in the recent past, is still challenging due to the lack of an atomic level understanding of the underlying signal-relay principles. Utilizing the small, not-allosterically regulated, lipase A from *Bacillus subtilis* (BsLA) as model protein, we show that sequence-based methods which capture the evolutionary coupling (see ref. 28 and references therein) between residues in a protein family can yield valuable information about the functional importance and hence potential modes of information flow within proteins (Fig. 1a). So far, those bioinformatic predictions have only been in rare cases experimentally validated by alanine-scanning^48^ or site-saturation mutagenesis^49^. The here presented site-saturation mutagenesis data for BsLA (Fig. 1b) shows that both computational predictions and the experiment essentially yield similar results. Both evolutionary-coupling analyses and site-saturation scanning mutagenesis identified a stretch of residues at the N-terminus of BsLA as functionally important (“mutationally sensitive”) and evolutionary coupled (Fig. 1), highlighting the complementarity of both methods. Based on this data, the N-terminus of BsLA was chosen as the most promising site for fusion of the CitAP sensory PAS domain expected to result in perturbation of BsLA function by ligand-binding induced conformational changes in the sensory domain. The presented strategy yielded a well folded artificial two-domain enzyme (CitAP-BsLA), whose function could readily be controlled by citrate binding in the fused sensory domain. Hereby, CitAP-BsLA showed decreased activity with increasing citrate concentrations (Fig. 2b). When purified CitAP-BsLA is stored for extended periods of time at 20 °C, proteolytic cleavage of the two domains is observed (Supplementary Figure 11). In consequence, in samples stored for 9 days at 20 °C, the covalent linkage between the CitAP-PAS and BsLA domains is to an large extend broken and the corresponding functional response is abolished (Supplementary Figure 12). This observation provides additional evidence for signal-relay between the citrate binding CitAP-PAS domain and BsLA. With respect to the mechanism of inactivation, studies using the detergent TX100 suggested that citrate represents an indirect modulator of CitAP-BsLA inhibition by TX100 rather than a direct allosteric inhibitor (Fig. 2c).

Using dose-response data, an apparent K_D_ for citrate of 32 ± 8 μM and a Hill coefficient *n*_*H*_ of 0.94 ± 0.11 were determined for CitAP-BsLA, both indicative of specific non-cooperative binding. Hereby, the K_D_ value of CitAP-BsLA is slightly larger than the one of the isolated CitAP sensor domain, for which a K_D_ of 11.1 at pH 8.0 was determined by isothermal titration calorimetry^37^. This discrepancy could for example arise from an altered citrate accessibility of the CitAP-PAS domain in the fusion protein, i.e. by a direct interaction between the two domains or by modulation of the quality or magnitude of the conformational change that is induced by citrate binding to the sensor CitAP PAS domain. Complementary, studies using different citrate analogs revealed a high specificity of the ligand-binding controlled enzyme with very similar properties as the isolated CitAP sensor domain^37^. Thus, CitAP-BsLA clearly represents an example of a designed artificial, highly active, yet very specific ligand-binding controlled enzyme.

**Figure 6:**
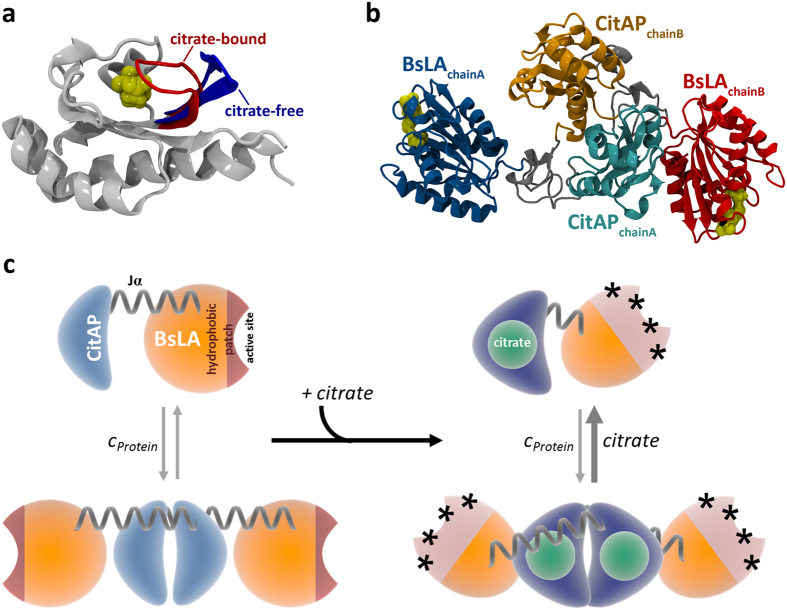
(**a)** Citrate-bound and citrate-free structure of the CitAP-PAS domain, as used for model-building. The citrate-bound structure (PDB ID: 2J80) is shown in grey, with the closed lid, containing the minor loop (residues 99–104), colored in red. Citrate is depicted as yellow van der Waals (vdW) surface. The corresponding citrate-free structure was obtained from a 100 ns MD simulation of the citrate-bound structure. For clarity, only the opened lid is shown (colored in blue). (**b)** Best model obtained from MD simulations. The BsLA and CitAP-PAS domains of the dimer are colored as in Fig. 5c. The catalytic triad of the lipase is shown in yellow. (**c)** Schematic illustration of a potential mechanism for the regulation of BsLA activity. The citrate-induced rearrangement of the CitAP-PAS and BsLA domains relative to each other results in the exposure of additional hydrophobic surface patches (marked by asterisks) which allows for increased binding of Triton-X100 to BsLA, which acts as an inhibitor of BsLA.

The membrane-bound SHK CitA that constitutes the sensory receptor of the CitA/CitB two-component system (TCS) of *K. pneumoniae*, is responsible for induction of citrate fermentation genes under anoxic conditions in the presence of environmental citrate^50^. Citrate-binding to the periplasmic CitAP PAS domain constitutes the trigger for structural-changes within the sensory domain that are transmitted *via* the connecting TM2 helix to the effector HK, eventually leading to quaternary structural changes within the CitA dimer which probably influences HK autophosphorylation^46^. Based on nuclear magnet resonance (NMR) spectroscopic and X-ray data, obtained for the citrate-free and citrate-bound form of the isolated CitAP PAS domain, it was suggested that citrate-binding to CitAP PAS results in closing/bending of the PAS β-scaffold by a rearrangement of the minor (residues 99–104) and major loop (residues 68–90)^46^. Moreover, the citrate-free structure of CitAP PAS lacks electron density in the surface exposed major loop, indicative of increased flexibility^46^. This hypothesis is fully corroborated by our 100 ns MD simulation of the citrate-free form of the isolated CitAP-PAS domain, where we observed a large-scale rearrangement of surface exposed loops of the citrate binding site and a stretching/flattening of the central β-scaffold in the absence of bound citrate (Fig. 6a; Supplementary Figure 6). In terms of global structure, it is assumed that full-length CitA possesses an elongated parallel dimeric structure with gross structural similarity to other soluble PAS domain containing SHKs like bacteriophytochromes^51^ or the artificial light-dependent HK YF1^42^. According to our simulations and SAXS data, it is unlikely that CitAP-BsLA adopts such an elongated parallel dimer structure. In the best model obtained from MD simulations and SAXS envelope reconstructions, we observed a dimeric arrangement of the CitAP-PAS domain with the BsLA domain being arranged parallel to the CitAP-PAS dimer flanking the sensory module on both sites (Figs 5c and 6b). The catalytic triad of BsLA is accessible in both subunits of the dimer as well as in both monomer models (Fig. 6b), enabling robust lipolytic activity of the fusion protein. While we believe that the overall subunit arrangement revealed by the SAXS-guided MD simulations is physically feasible, detailed structural questions cannot be addressed using the present model. In particular, the citrate-induced structural changes of CitAP-BsLA appear globally too subtle to be modelled accurately from SAXS data, since both the MD-derived models as well as the SAXS envelopes of the dimer in the citrate-free and citrate-bound form are very similar. To better understand the mechanism of the citrate-dependent functional response of CitAP-BsLA a number of complementary biochemical and biophysical techniques were used, which together hint at global tertiary/quaternary structural changes associated with citrate binding and hence ligand-binding dependent control of CitAP-BsLA. Several mechanistic scenarios could account for the observed citrate-dependent modulation of the TX100 inhibition of CitAP-BsLA. Based on our data, the most likely explanation is a small-scale structural rearrangement of the two domains relative to each other (illustrated in Fig. 6c), which would by congruent with the observed differences in Trp/Tyr fluorescence, interpreted as different Tyr/Trp FRET efficiencies in the presence and absence of citrate (Fig. 3a) as well with the small change in compactness of the molecule observed by AUC and SAXS (Table 2). This rearrangement results in the exposure of additional hydrophobic surface patches (marked by asterisks in Fig. 6c), as evidenced by bis-ANS binding studies (Fig. 3c,d) and an decreased K_D_ for Triton X-100 in the presence of citrate (Fig. 2c), which allows increased binding of the non-ionic detergent TX100 facilitating increased inactivation of the BsLA domain in the presence of citrate. Likewise, this rearrangement could impose strain to the BsLA structure relayed by the Jα-linker to first β-strand (β3) of the BsLA domain, which was identified by our computational and mutagenesis studies as mutationally sensitive (Fig. 1), onto the active site, in turn inactivating the enzyme. However, based on current data and without a crystal structure of the fusion protein, it is impossible to delineate between these scenarios.

In conclusion our study highlights the complementarity of evolutionary coupling analyses and site-saturation mutagenesis in identifying functionally important residues and potential pathways of information flow within proteins. As exemplified here for a small bacterial lipase, this information can be exploited for the construction of artificially controlled multidomain proteins. The simplicity of the here employed fusion strategy poses the interesting question if the catalytic mechanism of some enzymes is evolutionary optimized in a way that allows it to be easily perturbed by small conformational changes and/or non-natural protein-protein interactions. Such an evolutionary design could easily be realized by domain fusion and could account for the ubiquitous presence of allostery and multidomain sensory receptors.

## Methods

### Molecular biological and microbiological methods

Details about general molecular biological methods, site-saturation mutagenesis, fusion protein construction, expression of gene fusions and protein purification can be found in the Supplementary Materials.

### Evolutionary coupling analysis

Evolutionary coupling analysis was carried out for lipases using the BsLA sequence (Uniprot ID: P37957) as input sequence for the EVcouplings webserver (www.evfold.org). For the generation of the alignment the JackHHMer software (5 interations)^52^, implemented as part of the EVcouplings webserver, was utilized, to search the Uniprot database^53^ for sequences similar to BsLA. We ran an unrestrained search not limiting the number of sequences in the alignment, which retrieved 149.524 sequences with an E-value cutoff of 10E-3, covering 168 out of 181 residues of the query BsLA sequence. In a subsequent restrained run we limited the number of sequences in the alignment to 20.000 while using the same E-value cutoff. This search produced an alignment containing 20.000 sequences covering 176 out of 181 residues of the query sequence. Covariation information was inferred employing the plmDCA (pseudolikelihood maximization for Potts models with direct coupling analysis algorithm)^54^, implemented in the EVcouplings webserver. Evolutionary constraints (EC) values were mapped onto the B-factor field of the BsLA X-ray structure (PDB ID: 1I6W) and visualized by using Pymol v1.7.0.0 (Schrödinger Inc., NY, USA).

### High-throughput lipase assay and determination of the mutational sensitivity of BsLA

BsLA was used in a previous study as a model protein to assess the full protein landscape towards ionic liquidresistance^34^ and detergent tolerance^33^. All variant genes were expressed in *E.coli* BL21(DE3) and fused to a PelB secretion signal, which led to an unspecific release into the culture supernatant. The mean activity against *p*-nitrophenylbutyrate (*p*-NPB) of 96 *E. coli* BL21(DE3) clones harbouring the pET22b(+) vector with no insert was used to determine the experimental background and standard deviation (σ). All variants with an activity lower than the mean of the experimental background ±3 σ were considered as inactive. The B-factor of the pdb file (PDB-ID: 1I6W) was replaced with the absolute number of inactive variants for each of the 181 BsLA amino acid positions to generate the representation shown in Fig. 1.

### Determination of citrate-dependent lipase activity

BsLA lipolytic activity was measured using *p*-NPB as the substrate at 37 °C. Activity measurements were carried out in 1 cm disposable cuvettes with 100 mM 3-(N-morpholino)propanesulfonic acid (MOPS) buffer, pH 7,5 supplemented with 50 μM Triton X-100 (TX100) as assay buffer. Substrate stock solutions were prepared in acetonitrile containing 16 mM *p*-NPB. A suitable volume of enzyme was pipetted into the cuvette placed into a Beckman DU650 UV/Vis spectrophotometer temperature controlled to 37 °C. Assay buffer was heated to 37 °C in a thermo-block. Immediately before the activity measurement, the assay buffer was mixed with the substrate stock solution to yield an assay substrate concentration of 0.8 mM. This mixture was vortexed briefly and then added to the enzyme solution in the cuvette. Hydrolysis of *p*-NPB was monitored by measuring the release of *p*-nitrophenolate (*p*-NP) at 410 nm over 60 seconds. The lipolytic activity of the constructs was calculated using the molar extinction coefficient of *p*-NP (15.000 M^−1^ cm^−1^). All measurements were carried out in triplicate. For the determination of the citrate-dependent lipolytic activity of BsLA and CitAP-BsLA sodium citrate was added to the reaction mixture in concentrations up to 10 mM. Dose-response curves were obtained by plotting the relative lipolytic activity against the logarithmic citrate concentration. Dose-response data was fitted using Origin 9 G employing a four parameter logistic dose-response model according to the following equation:


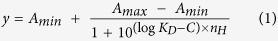


With A_max_ and A_min_ representing the top and bottom asymptotic activity values, *K*_*D*_ the apparent dissociation constant, *C* the citrate concentration and *n*_*H*_ the Hill slope.

### Determination of the effect of Triton-X100 (TX100) on the citrate-dependent activity of CitAP-BsLA

The TX100 dependence of the citrate-response of CitAP-BsLA was determined using the same experimental setup as described for the citrate-dependent lipase activity assay. The sodium citrate concentration was kept constant at 0 mM, 0.2.mM and 1 mM while the TX100 concentration was varied from 0 mM to 160 μM. All measurements were performed in triplicate and the data was analysed as described above.

### Tryptophan fluorescence

The fluorescence of aromatic amino acids was monitored in the presence and absence of 1 mM citrate for CitAP-BsLA and wild-type BsLA. For all measurements 1 cm quartz cuvettes (Hellma Analytics, Müllheim, Germany) were used employing a Cary Eclipse^TM^ spectrofluorimeter (Varian GmbH, Darmstadt, Germany) temperature controlled to 37 °C. A bandwidth of 5 nm was used in both the excitation and emission. CitAP-BsLA and wild-type BsLA were diluted to 3 μM in 10 mM glycin buffer pH 10 supplemented with 10 mM NaCl. Tryptophan fluorescence emission was measured from 300 nm to 400 nm while exciting the sample at 295 nm. When the sample is excited at 278 nm, both Trp and Tyr sidechains are excited and hence contribute to the observed fluorescence emission spectra which were recorded from 280 nm to 400 nm. The influence of citrate on the emission properties of the aromatic amino acids of the protein was determined by adding 1 mM of sodium citrate to the same protein sample.

### 4,4′-dianilino-1,1′-binaphthyl-5,5′-disulfonic acid (bis-ANS) fluorescence

Bis-ANS binding studies were carried out in 1 cm quartz cuvettes (Hellma Analytics, Müllheim, Germany) using a Cary Eclipse^TM^ spectrofluorimeter (Varian GmbH, Darmstadt, Germany) temperature controlled to 37 °C. Bis-ANS was dissolved in acetonitrile and added to protein solutions to a final concentration of 6 μM. Protein samples were diluted to 3 μM with 10 mM glycine buffer pH 10 supplemented with 10 mM NaCl. The influence of citrate on the emission properties of bis-ANS was determined by adding 1 mM of sodium citrate (dissolved in 10 mM glycine buffer pH 10 supplemented with 10 mM NaCl) to the sample containing the dye and the respective protein. Bis-ANS emission spectra were recorded from 400 nm to 600 nm by exciting the dye at 385 nm (emission and excitation band-width: 5 nm).

### Circular dichroism (CD) spectroscopy

Far-UV circular dichroism (CD) spectra were recorded using2 mm quartz cuvettes (Hellma Analytics, Müllheim, Germany) using a JASCO J-810 spectropolarimeter temperature controlled to 37 °C. All protein samples were diluted in 10 mM glycine buffer (pH 10) supplemented with 10 mM NaCl to a final concentrations of 0.1 mg/ml (approx. 3 μM). CD spectra were collected between 190 and 250 nm in 1 nm intervals with a scan speed of 50 nm/min. Ten spectra were averaged to obtain the final CD spectrum of the respective sample. The influence of citrate on the far-UV CD spectra of CitAP-BsLA was determined by adding 1 mM sodium citrate (dissolved in 10 mM glycine buffer pH 10 supplemented with 10 mM NaCl) to the protein sample. Additionally the influence of 50 μM Triton X-100 was tested. Near-UV CD spectra were recorded from 250 nm to 370 nm using the same setup. Samples were diluted to a final concentration of 1 mg/ml (approx. 30 μM) using 10 mM glycine buffer (pH 10) supplemented with 10 mM NaCl.

### Analytical ultracentrifugation (AUC)

Freshly thawed CitAP-BsLA solutions at a concentration of 0.5 mg/ml dissolved in 10 mM glycine buffer pH 10 supplemented with 10 mM NaCl (±citrate) were filled into custom-produced titanium centerpieces with sapphire windows and optical pathlengths of 20 mm. Upon inserting the cells into the rotor, optical alignment along the centrifugal field is ensured by the application of a custom-made cell alignment tool (Nanolytics). Sedimentation velocity experiments were carried out on a BeckmanCoulter XL-A/XL-I Analytical Ultracentrifuge using absorbance optics (l = 275 nm) at 25 °C and an angular velocity of 40 krpm. The data were analyzed with the standard c(s) model in SEDFIT version 12.5 (https://sedfitsedphat.nibib.nih.gov/software/default.aspx) using Bayesian prior expectations for weighting the regularization. Buffer density and viscosity were calculated incrementally using Sednterp 2.0 according to the given composition. Likewise, the partial specific volume (0.734 mL/g) was calculated incrementally according to the amino acid composition. After completing a conventional c(s) analysis with uniform prior, the c^(Pδ)^(s) distributions were calculated as a secondary analysis, based on the prior expectation that the protein sample exclusively contains monodisperse species resulting in sharp peaks^55^. Two major peaks (monomer and dimer) as well as up to two minor peaks representing higher oligomers were automatically detected from an existing c(s) distribution. For each, a numerical representation of a delta-peak (width = 0.1 S) is placed at the weight-average s-value integrated across the peak. From this c^(Pδ)^(s) distribution the relative peak concentrations were calculated. Since no material outside the peaks was assigned by the c^(Pδ)^(s) distribution, the validity of the prior expectation is demonstrated. The corresponding frictional ratios (f/f_0_) are related to the diffusion coefficient and were calculated from the respective sedimentation coefficient and the molecular mass of the species using the Svedberg equation. All plots of AUC raw data, best fits and residuals were created with the software GUSSI, which can be downloaded from the MBR Software Page (http://biophysics.swmed.edu/MBR/software.html). Data plots of c(s) and c^(Pδ)^(s) distributions were created by in-house developed software.

### Small angle X-ray scattering (SAXS)

SAXS was measured of CitAP-BsLA (0.5 to 5.0 mg/mL, 10 mM glycine buffer pH 10, 10 mM NaCl (±citrate), 10 °C sample temperature) at the beamline BM29 at the ESRF^56^. Measured data were scaled by the concentration. The excluded Porod volume was calculated with the program DATPOROD and the molecular mass was estimated by using the reported protein density of 0.588 g/mL^47^. The distance distribution function *P*(*r*) was determined using the program DATGNOM. In total 20 *ab initio* models were generated using the program DAMMIF, averaged and the filtered model was used. The envelope function was determined using the SITUS package^57^.

### CitAP-BsLA model building and molecular dynamic (MD) simulations

The detailed strategy for modelling of the dimeric CitAP-BsLA complex is summarized in the Supplementary Materials. CitAP-BsLA monomer models were built with the program BUNCH^58^ of the ATSAS package^47^. In all cases, template coordinates were taken from the PDB structures with IDs 2J80^46^ (CitAP) and 1I6W^30^ (BsLA). The linker connecting the CitAP and BsLA domains in the monomer, the His6 tag and all other remaining missing residues of the fusion protein were modelled as Cα traces during the fitting procedure. Afterwards, the Cα traces were extended to all-atom models with the web server MaxSprout^59^. As the all-atom extension of prolines failed, these were modelled by a superposition of a template proline residue onto the proline backbone obtained from MaxSprout. Dimer models were either built manually, by superimposing the corresponding monomer models onto the dimeric crystal structure of CitAP-PAS (PDB-ID 2J80), or were assembled *ab initio* by oligomerizing the monomer models using the program SASREF^58^ optimizing the dimer orientation against SAXS data at high protein concentration (100% dimer). Further details are given in the Supplementary Material and Supplementary Table 2. The quality of all models was evaluated with the program CRYSOL^60^. CRYSOL computes theoretical scattering curves and compares these to the experimental data. As quality indicator for each model the χ values computed by CRYSOL were used, which present a measure for the discrepancy between theoretical and experimental curves. In order to improve the initial models, a 100 ns molecular dynamics (MD) simulation was performed for each dimeric assembly and a theoretical scattering curve was calculated for every 200 ps snapshot of each trajectory and fitted against the experimental data using CRYSOL. Details can be found in the Supplementary Materials.

## Additional Information

**How to cite this article**: Kaschner, M. *et al*. A combination of mutational and computational scanning guides the design of an artificial ligand-binding controlled lipase. *Sci. Rep.* **7**, 42592; doi: 10.1038/srep42592 (2017).

**Publisher's note:** Springer Nature remains neutral with regard to jurisdictional claims in published maps and institutional affiliations.

## Supplementary Material

Supplementary Information

## Figures and Tables

**Table 1:** Comparison of the relative oligomer distribution of CitAP-*Bs*LA samples with (+) and without (−) 1 mM sodium citrate derived from analytical ultracentrifugation (AUC) and small-angle X-ray scattering (SAXS) data obtained for CitAP-*Bs*LA at low concentration (0.5 mg/ml).

	relative oligomer distribution			
	AUC^$,§^		SAXS^&^	
−	+	−	+	
[%]	[%]	[%]	[%]	
Monomer	73	85	73	86
Dimer	23	12	27	14
>Dimer	4	3	n.d	n.d

^$^For AUC experiments, the relative oligomer distribution was estimated using Bayesian statistics assuming the presence of discrete species of known molecular mass. ^&^From SAXS experiments the average molecular mass was determined, which corresponds directly to the average molecular mass of a population between monomer and dimer with known molecular mass. The molecular mass was determined from the concentration normalized forward scattering and the excluded volume multiplied by the protein density, and the average molecular mass is given. Theoretical molecular mass: monomer: 38.5 kDa, dimer: 77 kDa; ^§^Values represent the mean of two independent sedimentation velocity runs, with an experimental error below 5%.

**Table 2:** Comparison of analytical ultracentrifugation (AUC) and small-angle X-ray scattering (SAXS) data for CitAP-*Bs*LA samples with (+) and without (−) 1 mM sodium citrate.

	sedimentation coefficent (AUC)^§^		Guinier Radius *R*_*g*_ (SAXS)^&^		frictional ratio (AUC)^§^		*D*_*max*_ (SAXS)^&^	
	−	+	−	+	−	+	−	+
	[S]	[S]	[nm]	[nm]	[f/f0]	[f/f0]	[nm]	[nm]
Monomer^$^	3.53	3.61	2.94	2.81	1.15	1.12	10.1	9.7
Dimer^$^	4.49	5.15	3.37	3.44	1.37	1.25	11.1	11.6
>Dimer	6.65	8.36	n.d	n.d	n.d.	n.d.	n.d	n.d

^$^Molecular mass: monomer: 38.5 kDa, dimer: 77 kDa; n.d. not detected; ^&^*Rg* and *Dmax* were determined from SAXS measurements at 0.5 and 5 mg/mL. At low concentration, the determined parameters primarily inform about the structural properties of the monomer, which is the predominwant population at that concentration. At high concentration the dimer is the prevalent species and the structural parameters inform primarily about the properties of the dimer. Dmax and Rg were determined from the distance distribution in real space. ^§^Values represent the mean of two independent sedimentation velocity runs, with an experimental error below 5%.
