# Supplementary material for: A combination of mutational and computational scanning guides the design of an artificial ligand-binding controlled lipase
Source: Sci Rep. 2017 Feb 20;7:42592. doi: 10.1038/srep42592 (PMC5316958; doi:10.1038/srep42592)
Supplement: Supplementary Information [file srep42592-s1.pdf]

Supplementary Materials

A combination of mutational and computational scanning guides the design of an artificial ligand-binding controlled lipase

Marco Kaschner, Oliver Schillinger, Timo Fettweiss, Christina Nutschel, Frank Krause, Alexander Fulton, Birgit Strodel, Andreas Stadler, Karl-Erich Jaeger and Ulrich Krauss*

	Content	Page
Supplementary Methods	General molecular biological and microbiological methods<br>Molecular dynamics simulations	2-5
Supplementary Table 1	Bacterial strains and plasmids used in this study.	6
Supplementary Figure 1	Evolutionary-coupling analysis of BsLA	7
Supplementary Figure 2	Heatmap of inactive BsLA variants	8
Supplementary Figure 3	Tryptophan fluorescence of CitAP-BsLA and wild-type BsLA.	9
Supplementary Figure 4	Sedimentation velocity analysis of CitAP-BsLA with and without citrate.	10
Supplementary Figure 5	SAXS data obtained for CitAP-BsLA.	11
Supplementary Methods	Computational modelling and molecular dynamics simulations of the CitAP-BsLA complex	12
Supplementary Table 2	Summary of the model-building strategy	13
Supplementary Figure 6	RMSD of the CitAP PAS major loop minor loop during MD simulations of isolated CitAP	14
Supplementary Figure 7	Structural changes during the MD simulations of the models of the fusion protein dimer.	15
Supplementary Figure 8	Time evolution of χ during the MD simulations of the four fusion protein models.	16
Supplementary Figure 9	Models of the fusion protein dimer at the end of the MD simulations.	17
Supplementary Figure 10	Triton-X100 dependency of the citrate dependent activity response of CitAP-BsLA and BsLA	18
Supplementary Figure 11	Proteolytic stability of purified CitAP-BsLA during long-term storage of the protein	19
Supplementary Figure 12	Citrate dependent response of a CitAP-BsLA sample stored for up to 9 days at 20 °C	20
Supplementary Table 3	Deconvolution of CitAP-BSLA far-UV CD data	21
Supplementary Table 4	Comparison of the CD predicted secondary structure content and the secondary structure content derived from DSSP analysis of CitAP-BSLA component structures.	22
Supplementary References		23

Supplementary Methods

Chemicals. If not mentioned otherwise, all chemicals were purchased from Carl Roth GmbH (Karlsruhe Germany) or Sigma-Aldrich (St. Louis, MO, USA) in highest purity available.

General Molecular Biological techniques. Isolation of recombinant plasmids, gel extraction of DNA fragments, DNA ligation, and transformation into *E. coli* strains were carried out according to standard laboratory protocols [1].

Whole-protein site-saturation scanning mutagenesis. A full library containing every possible single-point amino-acid substitution of BsLA was generated using a statistically optimized two-stage procedure [2]. The results of this mutagenesis and a detailed description of the experimental set up have been described previously [3].

Construction of gene fusions. All plasmids used in this study are shown in Supplementary Table 1. The CitAP-BsLA gene fusion was constructed by subcloning a synthetic gene fragment coding for the isolated CitAP domain into a BsLA containing expression vector. The periplasmic CitA PAS domain (CitAP) DNA fragment was obtained as a synthetic gene fragment (Eurofins Genomics, Ebersberg, Germany) (pCR2.1-CitAP). The pCR2.1-CitAP vector was hydrolyzed using *Nde*I and *Hind*III restriction endonucleases. The resulting CitAP gene fragment was subsequently cloned into a similarly hydrolyzed pET28a-based BsLA expression vector (pET28a-nLOV-BsLA) which already contained a gene fusion consisting of the YtvA-LOV gene region and the full-length BsLA encoding gene [4]. Please note that, by hydrolyzing pET28a-YLOV-BsLA with *Nde*I and *Hind*III, the YtvA-LOV domain encoding segment is released enabling substitution of the LOV domain encoding gene fragment with CitAP PAS. Using this strategy a tripartite gene fusion consisting of the CitAP PAS domain

(residues 45 to 177 of full-length CitA), an YtvA Jα-linker segment (residues 132 to 147 of full-length YtvA) and the BsLA encoding gene (residues 1 to 181 of full-length BsLA) was constructed. Additionally, a cleavage site for the Factor Xa protease was introduced between the YtvA Jα linker and the first amino acid of the BsLA domain (amino acid sequence: IEGRE). Before heterologous expression, all constructs were verified by sequencing (SeqLab GmbH, Göttingen, Germany). Additionally, all gene fusions contained an expression vector derived N-terminal His6-Tag (amino acid sequence: MGSSHHHHHHSSGLVPRGSH) to enable the easy purification of the recombinant protein from *E. coli* by immobilized metal affinity chromatography (IMAC).

Bacterial strains and plasmids. All strains and plasmids used in this study are listed in Supplementary Table 1. All strains were grown either in Luria-Bertani (LB) broth (Carl Roth®, Arlesheim, Switzerland) or in autoinduction (AI) media (adapted from Studier et al. 2005 [5] for heterologous production of recombinant proteins. In brief, AI medium contained 15 g/L casein hydrolysate, 0.5 g/L glucose, 30 g/L yeast extract and 6,25 g/L glycerol in 100 mM potassium phosphate buffer pH 7 and 2 g/L lactose added for induction. Glucose and lactose solutions were autoclaved separately as stock solutions (50 g/L glucose, 20 g/L lactose) and mixed with the rest of the medium immediately before use. For maintenance of plasmids 50 µg/ml kanamycin was added to all media. CitAP-BsLA and wild-type BsLA were expressed in *E. coli* BL21(DE3) by using an overnight seed culture grown in LB medium (37 °C, 120 rpm) to inoculate 1 L of AI medium in 5 L Erlenmeyer flasks to an OD_{600nm} of 0.05. The cultures were grown at 37 °C for 2 hours at constant agitation (120 rpm) and then shifted to 15 °C and grown for another 72 hours.

Protein purification. CitAP-BsLA and wild-type BsLA were produced in *E. coli* BL21(DE3) as described above. The cell pellet (5 g cells, wet weight) was dissolved in 25 ml lysis buffer

(50 mM sodium phosphate buffer pH 8, supplemented with 300 mM NaCl and 10 mM imidazole). Cells were lysed by passing the cell-suspension 3-times through a chilled French Pressure Cell (ThermoScientific, Waltham, USA) at a constant pressure of 1100 bar. The soluble fraction was separated from cell debris by centrifugation for 20 minutes at 38465 x g at 4 °C. The lysate was purified by immobilized metal affinity chromatography using a Superflow Ni-NTA resin (QIAGEN, Hilden, Germany). All purification steps were carried out at room-temperature employing an ÄKTApurifier FPLC system (GE Healthcare, Buckinghamshire, UK) fitted with a XK16/20 column. The column was equilibrated with lysis buffer. The crude cell-free extracts were loaded using a 50 ml Superloop^{TM} (GE Healthcare, Buckinghamshire, UK) and unspecifically bound proteins were removed by washing the column with 50 mM sodium phosphate buffer pH 8, supplemented with 300 mM NaCl and 20 mM imidazole. Elution of the His6-tagged target proteins was performed in 50 mM sodium phosphate buffer pH 8, supplemented with 300 mM NaCl and 250 mM imidazole. The purity of the eluted fractions was evaluated densitometrically by SDS-PAGE. Pure fractions were pooled and the buffer was exchanged to 10 mM Glycin pH 10 supplemented with 10 mM NaCl using a Sephadex^{TM} G25 (GE Healthcare, Buckinghamshire, UK) column. Desalted protein samples were concentrated using either Vivaspin20 (Sartorius, Göttingen, Germany) centrifugal concentrators or Vivacell (Sartorius, Göttingen, Germany) pressure cells employing a molecular weight cutoff of 10.000 Da. Concentrated samples were frozen in liquid nitrogen and stored at -20 °C until further use. For analytical ultracentrifugation (AUC) and small angle X-ray scattering (SAXS) analyses, CitAP-BsLA was further purified by preparative size-exclusion chromatography (SEC) by employing a Superdex^{TM} HR 10/30 column (GE Healthcare, Buckimhamshire, UK) and an ÄKTApurifier FPLC system at a constant flow-rate of 0.75 ml/min in 10 mM glycine buffer pH 10 supplemented with 10 mM NaCl.

Molecular dynamics (MD) simulations. All starting models (generated as described in the main manuscript) were energy minimized, equilibrated and subjected to 100 ns molecular dynamic (MD) simulations using Gromacs 5.0 [6]. Simulations were performed in the NTP ensemble at a temperature of 298 K and a pressure of 1 bar. The amber99sb-ildn-NMR force field [7] was used with the TIP3P water model [8] and sodium and chloride ions were added to neutralize the system and mimic a salt concentration of 10 mM. Force field parameters for citrate were taken from the general amber force field (GAFF) [9] using the program acpype [10]. Long-range electrostatics were treated with the PME algorithm [11]. Short range electrostatic and van der Waals interactions were computed up to a cut-off of 1.2 nm. To increase the time step length to 5 fs, virtual sites were introduced for all hydrogens [12]. As this cannot be done automatically for citrate, the simulations containing citrate did not use virtual sites and a time step of 2 fs was used instead. χ values were computed every 200 ps.

Supplementary Table 1. Bacterial strains and plasmids used in this study.

**Bacterial strain**	**Relevant genotype**	**Reference**
Escherichia coli DH5α	F– Φ80*lac*ZΔM15 Δ(*lac*ZYA-*arg*F) U169 *rec*A1 *end*A1 *hsd*R17 (r_K^-, m_K^+) *gal*$^-$ *pho*A *sup*E44 λ$^-$ *thi*$^-$1 *gyr*A96 *rel*A1	Invitrogen
Escherichia coli BL21(DE3)	F– *ompT gal dcm lon hsd* S_B(r_B^-, m_B^-) λ(DE3[*lac*I *lac*UV5-T7 gene 1 *ind*1 *sam*7 *nin*5])	Invitrogen
**Plasmid name**	**Relevant features**	**Reference**
pET28a	Km^R, P_{T7}, N-terminal His-tag, *lacI*	Novagen
pET28a-CitAP-BsLA	pET28a derivative, P_{T7}>CitAP-BsLA	this work
pET28a-BsLA	pET28a derivative, P_{T7}>BsLA	this work
pET22-BsLA	pET22b derivative, P_{T7}>BsLA	[13]

Evolutionary coupling analysis of BsLA

To further validate the evolutionary-coupling analyses presented in the main manuscript, a more restricted search was conducted, restricting the number of aligned sequences to 20.000, which marginally increased the alignment coverage from 168 (unrestrained run) to 176 out of 181 residues of BsLA. While the magnitude of the observed EC values differs between the two computations, the overall distribution of coupled residues remained largely the same (Supplementary Figure 1).

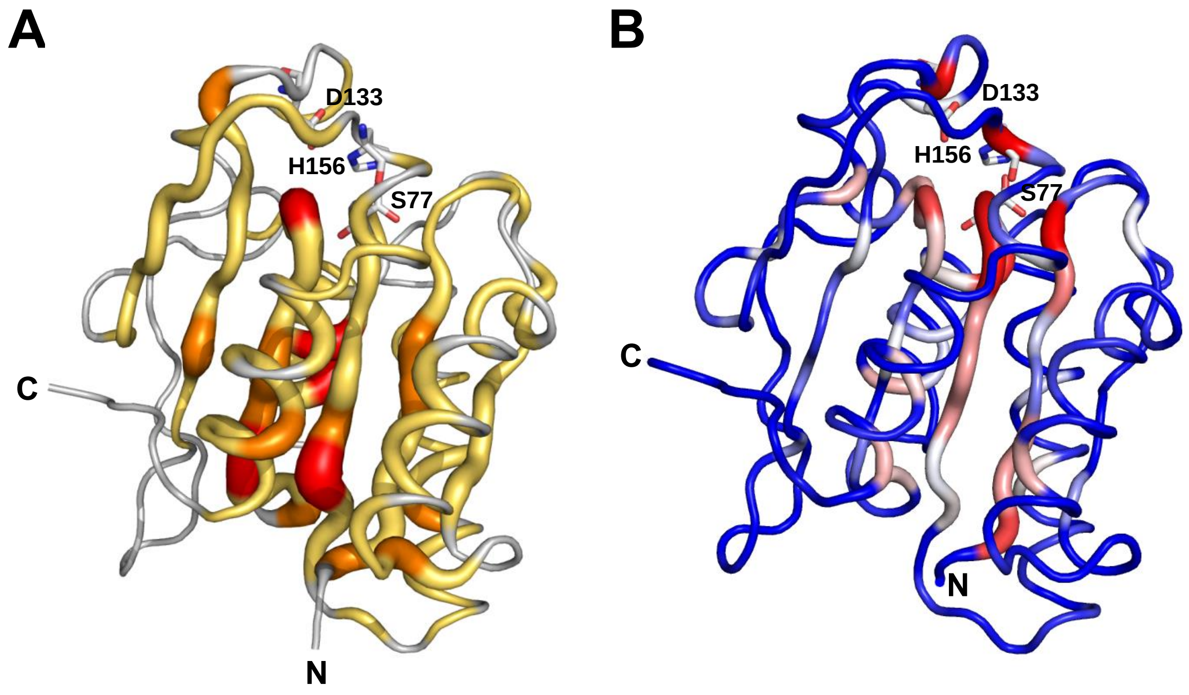

Supplementary Figure 1: Comparison of evolutionary-coupling analyses for a more restricted run (A) and site-saturation scanning mutagenesis data (B) mapped onto the X-ray structure of BsLA. Evolutionary coupled residues were inferred from a multiple sequence alignment containing 20.000 sequences (E-value cut-off 10E-3) using the EVcoupling webserver (www.evfold.org). The obtained EC scores were mapped onto the X-ray structure of BsLA (PDB Entry: 1I6W) [14]. The magnitude of the obtained EC scores is color coded (low values in yellow; high values in red). Additionally EC scores are encoded by sausage thickness representing the magnitude of the EC score. The number of inactive BsLA variants per residue was obtained from comprehensive whole protein site-saturation mutagenesis data (B) and mapped onto the BsLA X-ray structure. The fraction of inactive variants is encoded by color (blue: low values; red high values) and sausage thickness. The N- and C-terminus of BsLA are marked accordingly. The residues of the catalytic triad, Ser77, Asp133 and His156 are shown as sticks with oxygen in red, carbon in grey and nitrogen atoms in blue.

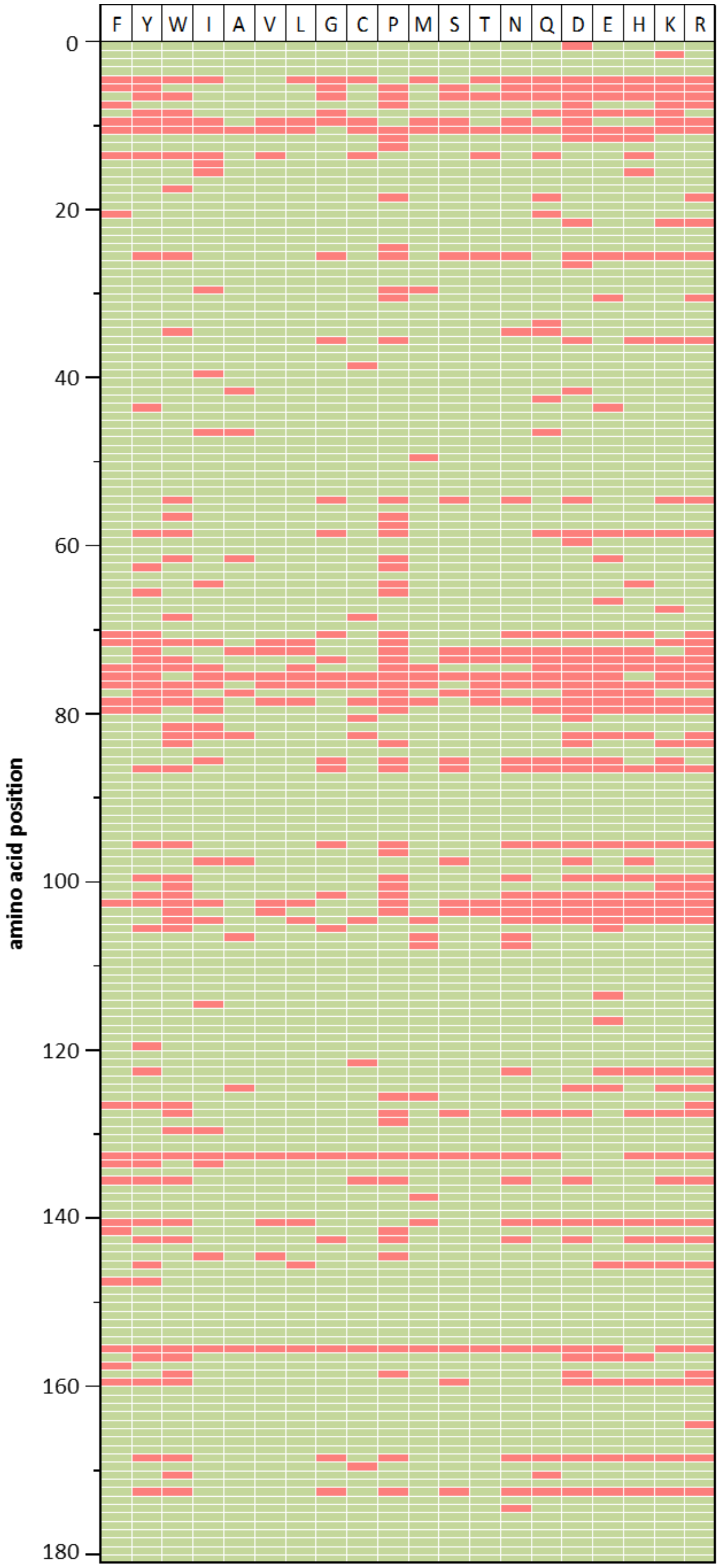

Supplementary Figure 2: Heatmap of inactive BsLA variants (red = inactive, green =active), Positions are indicated numerically and the substitutions by their amino acid abbreviation.

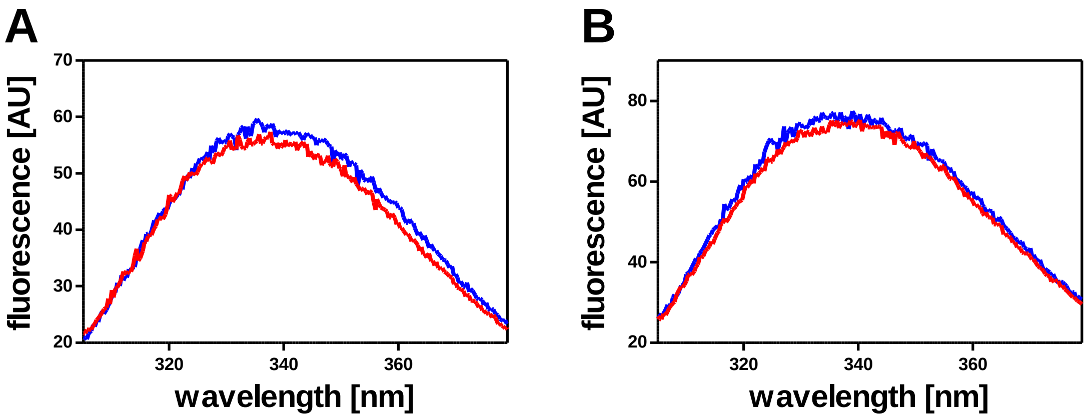

Supplementary Figure 3: Tryptophan fluorescence of CitAP-BsLA (A) and wild-type BsLA (B). Protein samples, diluted to 3 µM in 10 mM glycine buffer pH 10 supplemented with 10 mM NaCl, were excited at 295 nm. All fluorescence emission spectra were recorded in the presence (red line) and absence (blue line) of 1 mM sodium citrate.

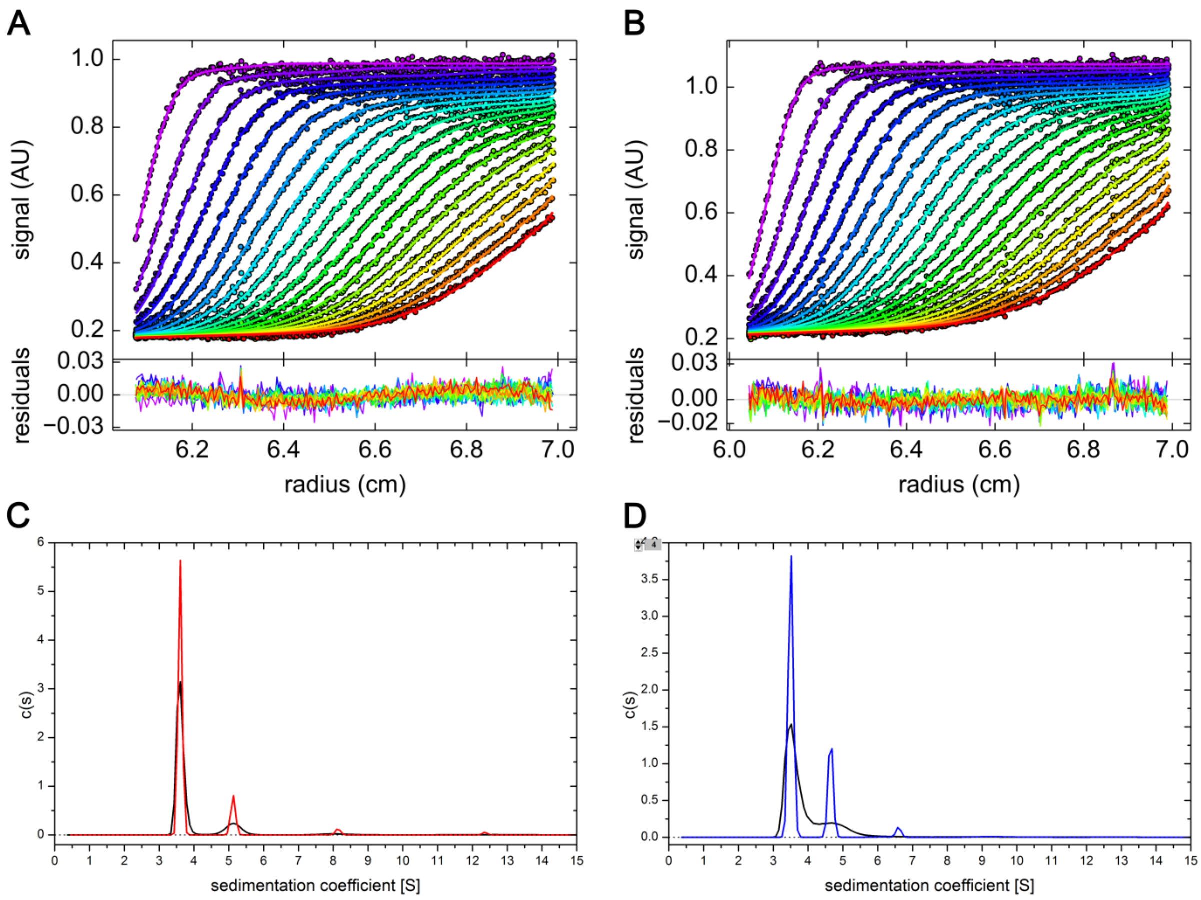

Supplementary Figure 4: Exemplary sedimentation velocity analysis of 0.5 mg/mL CitAP-BsLA solutions with (A and C) and without citrate (B and D), respectively, using absorbance detection. (A and B) The top panels show raw data (circles) and best fits (lines). For clarity, only every second scan of the data set is shown. The bottom panels display best fit residuals of the plotted scans. (C and D) The conventional sedimentation coefficient distributions c(s) are shown as black solid lines. In a secondary analysis, the $c^{(P\delta)}(s)$ distributions based on the prior expectation that each protein sample exclusively features monodisperse species are shown as red (with citrate) and blue lines (without citrate), respectively. The relative abundances of monomers and dimers are derived from the $c^{(P\delta)}(s)$ distributions.

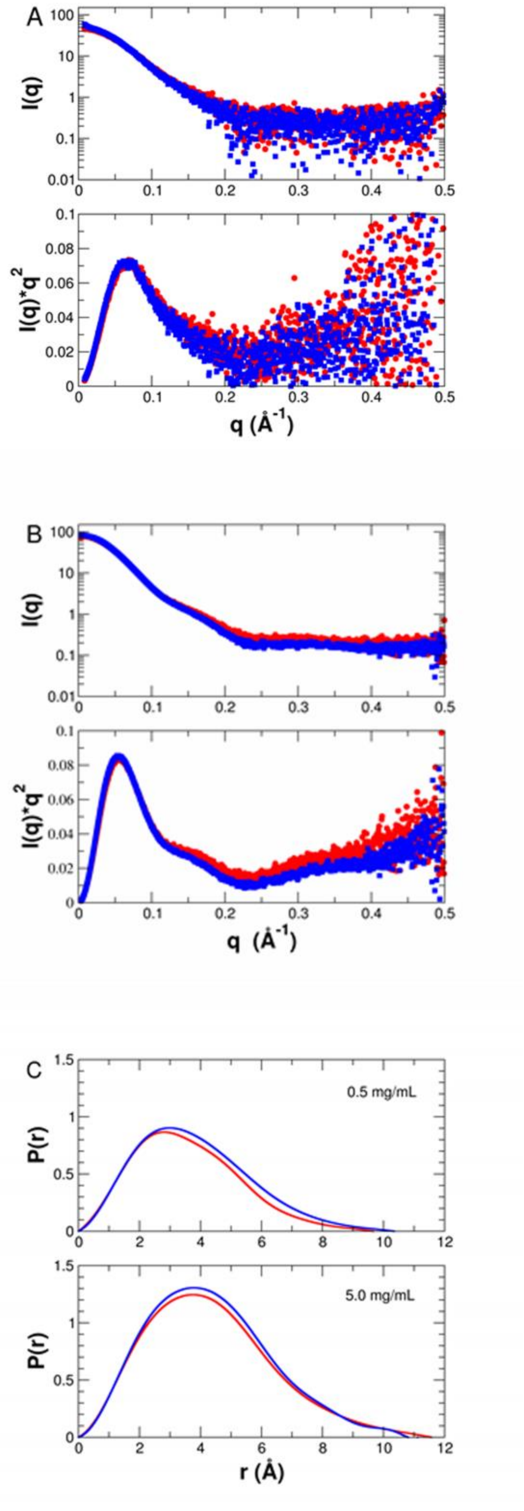

Supplementary Figure 5: SAXS data obtained for CitAP-BsLA. A) Scattering curves at low concentration (mainly monomer) and at B) high concentration (mainly dimer) with citrate (red line) and without citrate (blue line). (C) Distance distribution function P(r) derived from scattering data of CitAP-BsLA at 0.5 mg/ml and at 5.0 mg/ml. The P(r) function was derived from scattering data of samples of the indicated concentration with citrate (red line) and without citrate (blue line) by using the program DATGNOM of the ATSAS package [15].

Computational modelling and molecular dynamics simulations of the CitAP-BsLA complex

Initial monomer models were obtained from SAXS data at low concentration (0.5 mg/ml) where CitAP-BsLA is predominately monomeric. Monomer models were built using the program BUNCH of the ATSAS package [15] employing chain A of the citrate-bound CitAP-PAS structure (PDB-ID: 2J80) [16] and the monomeric BsLA structure (PDB-ID: 1I6W) [14] in the q-range from 0.01 up to 0.15 Å^{-1} for the citrate saturated and 0.017 up to 0.15 Å^{-1} for the citrate free solution. The constructed BUNCH models gave a good fit to the SAXS data with χ=1.06 for the citrate saturated and χ=1.12 for the citrate free protein solution. The R_g and D_{max} of the citrate-saturated model are 2.80 nm and 9.49 nm, and for the citrate free model 3.03 nm and 10.5 nm, respectively, which is in agreement with the measured experimental values. A dimeric assembly was subsequently generated by superimposing the BUNCH-derived monomer model onto the dimeric citrate-bound CitAP-PAS structure (PDB-ID 2J80) resulting in the model $M_{low\text{-}cit}$. To derive a model with a citrate-free CitAP-PAS conformation, we had to employ a CitAP-PAS structure which shows an open citrate-binding site. Unfortunately, the available citrate-free structure of CitAP-PAS (PDB-ID: 2V9A) [16], lacks electron density in the surface exposed major loop (residues 68 to 89 of CitAP-PAS) of the citrate-binding site [16], which was previously interpreted as increased flexibility of the respective loops, which are directly involved, and hence stabilized by binding of citrate [16]. Therefore, to obtain an all-atom model of citrate-free CitAP-PAS we used the closed (citrate-bound) CitAP-PAS structure (PDB-ID: 2J80), removed the citrate and performed a 100 ns MD simulation. During the simulation, the major loop of the CitAP-PAS domain shows increased mobility i.e. manifested as an increased RMSD over the trajectory (Supplementary Figure 6a). Moreover, the CitAP-PAS domain opens up and reaches a stable conformation with an open lid (minor loop movement) (see Figure 6a and Supplementary Figure 6), thus corroborating previous NMR and X-ray crystallographic studies of CitAP-PAS [16]. In contrast, the citrate-bound structure did not undergo any major structural rearrangements (Supplementary Figure 6). The lid of the corresponding open (citrate-free) CitAP-PAS model was subsequently transferred to the $M_{low\text{-}cit}$ model resulting in $M_{low\text{-}free}$. In addition, a set of two dimer models was generated using the corresponding CitAP-BsLA monomer models, assembling them using the program SASREF [15], which models oligomer complexes by optimizing the subunit orientation against experimental SAXS data, and then generating the open form of the CitAP-PAS domain as outlined above. This resulted in the models $M_{high\text{-}cit}$ and $M_{high\text{-}free}$, respectively.

Supplementary Table 2: Summary of the model-building strategy

**Model name**	**Protein concentration**	**Dimer/Monomer ratio (SAXS)**	**Citrate concentration**	**Citrate binding pocket**	**Rigid body fit program**
$M_{high\text{-}cit}$	high (5 mg/ml)	95:5	1 mM	bound/closed	SASREF + BUNCH
$M_{high\text{-}free}$	high (5 mg/ml)	98:2	0	free/open	SASREF + BUNCH
$M_{low\text{-}cit}$	low (0.5 mg/ml)	14:86	1 mM	bound/closed	BUNCH
$M_{low\text{-}free}$	low (0.5 mg/ml)	27:73	0	free/open	BUNCH

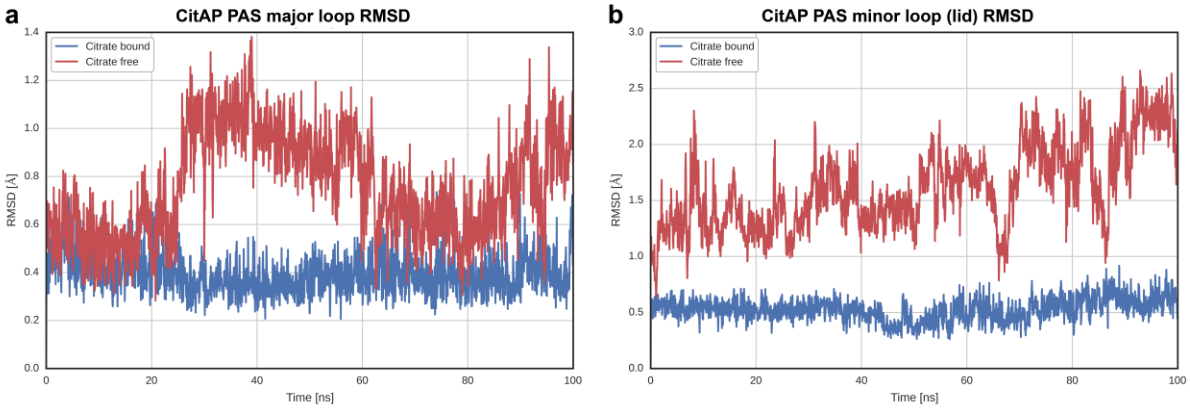

Supplementary Figure 6: (a) C_α RMSD of the CitAP PAS major loop (residues 68 to 89) and **(b)** minor loop (lid, residues 99-104) with respect to the mean positions during the citrate-bound (i.e. closed conformation) MD simulation. The trajectories were fitted to the closed crystal structure by minimizing the C_α RMSD of residues 12 to 94 and 109 to 128 (i.e. omitting residues in the opening lid and at the termini).

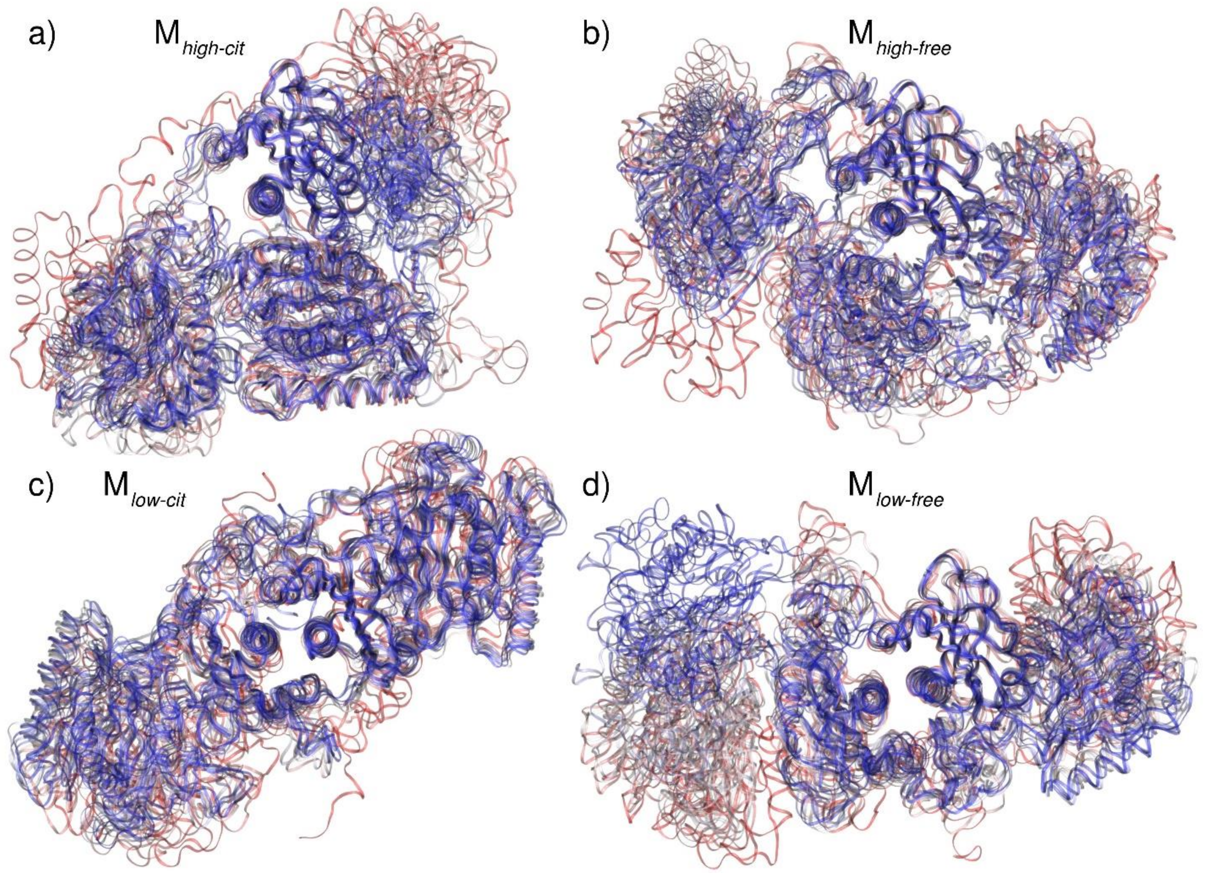

Supplementary Figure 7: Structural changes during the MD simulations of the models of the fusion protein dimer. The proteins are shown as ribbons and the colors represent structures at different times, changing from red at t = 0 ns to blue at t= 100 ns.

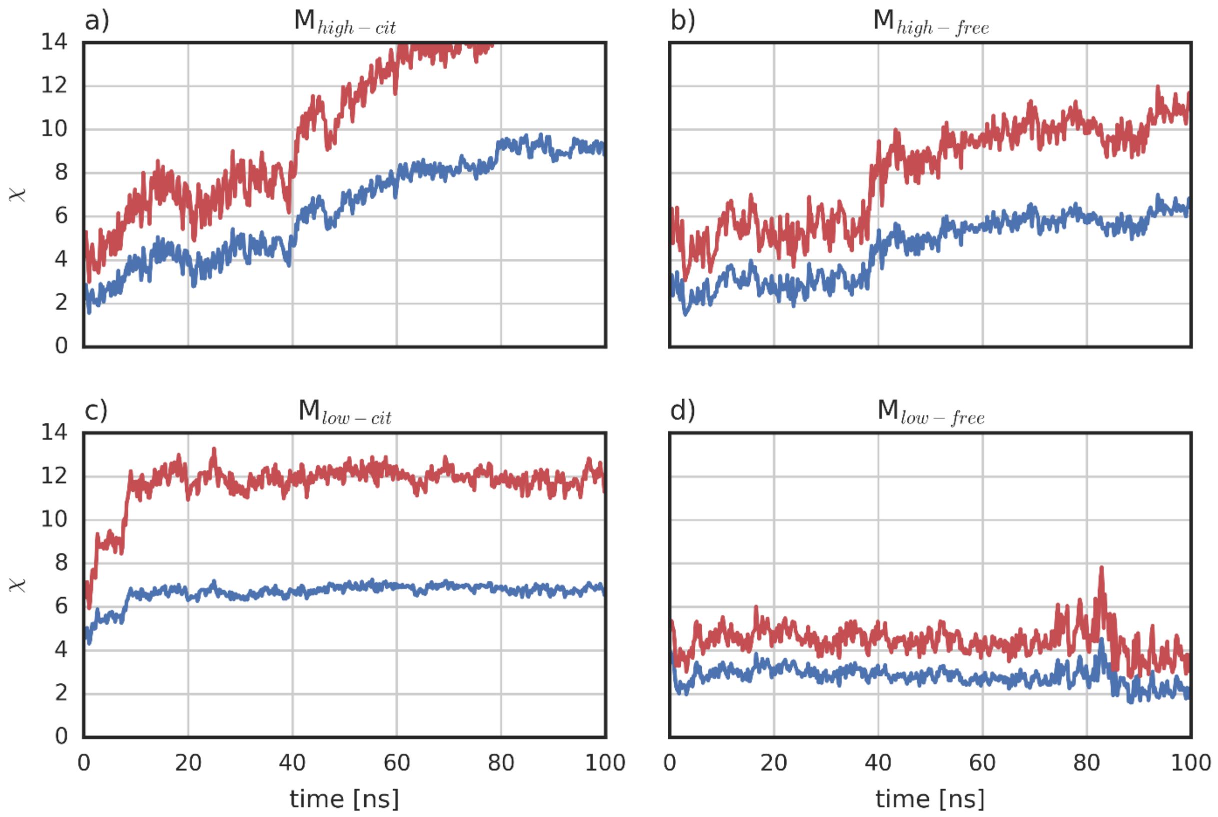

Supplementary Figure 8 Time evolution of χ during the MD simulations of the four fusion protein models. Deviation between the models and experimental data measured at high citrate concentration (blue) and without citrate (red) are shown. It should be noted that χ and not χ2 is presented as for the models in (a) – (c) χ2 can become large (> 100), making it difficult to present the results for all four models on the same scale.

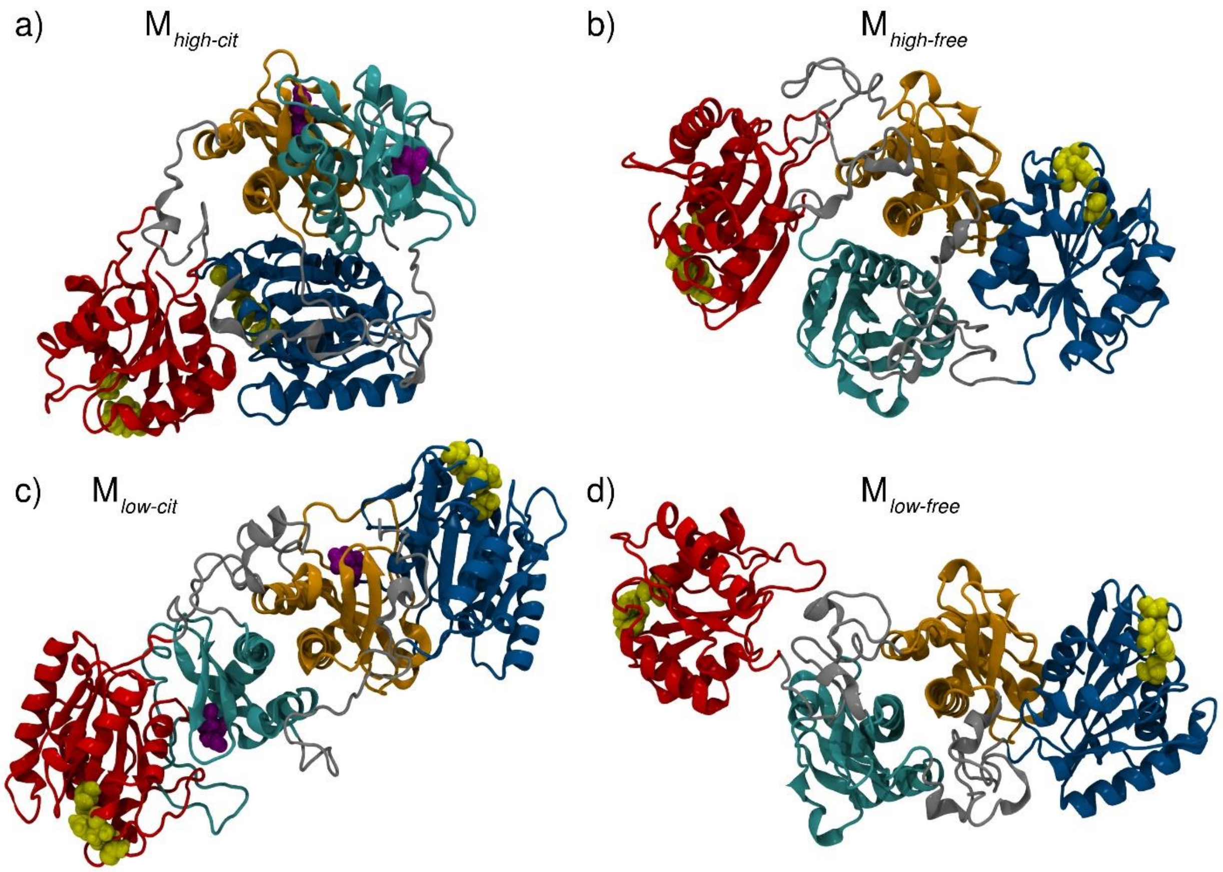

Supplementary Figure 9: Models of the fusion protein dimer at the end of the MD simulations. The proteins are represented in cartoon style with chain A being colored red (BsLA) and orange (CitAP), chain B is shown in blue (BsLA) and cyan (CitAP), and the linker and His-tag are colored gray in both chains. The BsLA residues forming the catalytic triad residues are represented as yellow van der Waals (vdW) surface, while in the models with citrate the ligand is shown as purple vdW surface.

Triton-X100 dependency of the citrate-dependent activity response of CitAP-BsLA and wild type BsLA

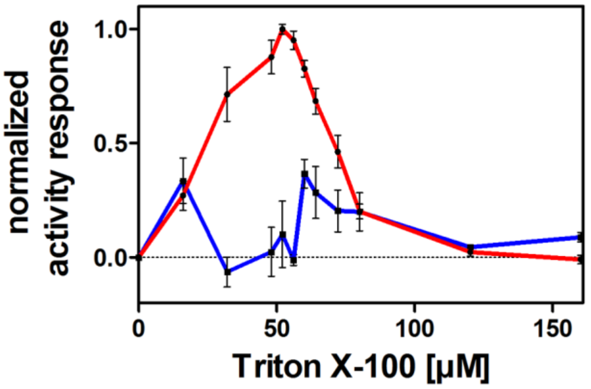

Supplementary Figure 10: Triton-X100 dependency of the citrate-dependent activity response of CitAP-BsLA (red line) and wild type BsLA (blue line). The data was normalized to the maximal acitivity response of CitAP-BsLA. Lipolytic activity was measured using *p*-nitrophenylbutyrate as substrate. Error bars depict the standard deviation of the mean derived from three independent measurements.

Proteolytic stability of purified CitAP-BsLA during long-term storage and citrate-dependent lipolytic activity of long-term stored CitAP-BsLA samples

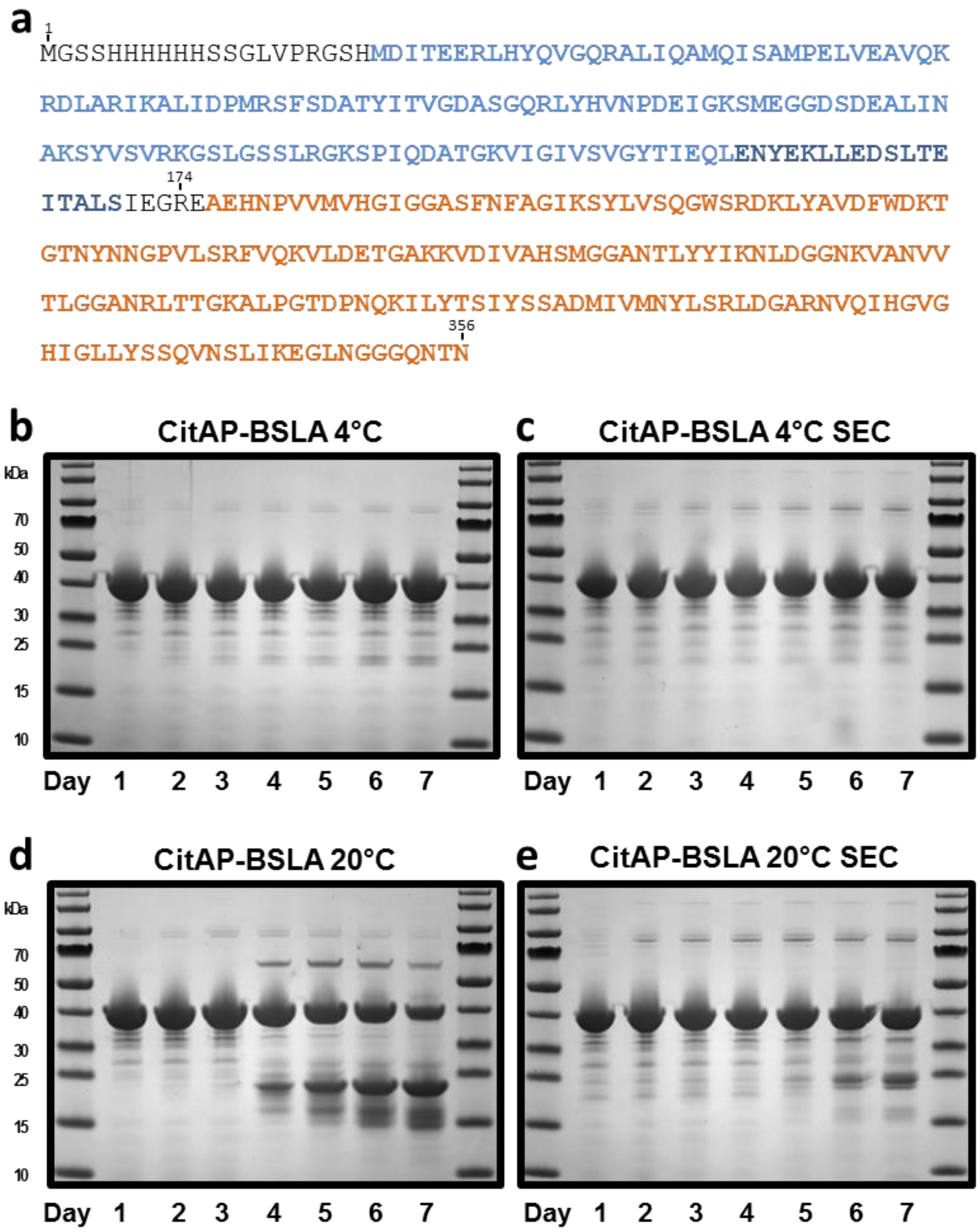

Supplementary Figure 11: Sequence of the fusion protein (a) and SDS-PAGE analysis of the proteolytic stability of purified CitAP-BsLA during long-term storage. In (a) the amino acid sequence of the CitAP-BsLA fusion protein is shown. The CitAP-PAS domain is marked in blue and the BsLA domain is highlighted in orange. The first and last amino acids are labelled, as is the position of the Factor Xa protease cleavage site (recognition sequence IEGRE). The panels (b-e) illustrate the proteolytic stability of the purified fusion protein under different storage conditions. Samples were either purified by immobilized metal ion affinity chromatography (IMAC) or IMAC followed by preparative size exclusion chromatography SEC) (b) IMAC purified CitAP-BsLA stored at 4 °C. (c) IMAC and SEC purified CitAP-BsLA stored at 4 °C. (d) IMAC purified CitAP-BsLA stored at 20 °C. (e) IMAC and SEC purified CitAP-BsLA stored at 20 °C.

In order to address the storage stability of purified CitAP-BsLA, we stored purified samples for 7 days under different conditions. Samples purified by immobilized metal ion affinity chromatography (IMAC) or samples purified by IMAC and preparative size-exclusion chromatography (SEC) were stored at either 20 °C or 4 °C. At defined time intervals a sample was withdrawn and analysed by SDS-PAGE (Supplementary Figure 11, b-e).

When the purified protein is stored at 20 °C, an additional lower molecular weight band (approx. 20 kDa) appears in SDS-PAGE analyses after about 4 days, which is accompanied by the reduction of the band corresponding to the fusion protein (approx. 38 kDa) (Supplementary Figure 11d). The effect is largely absent when the protein is stored at 4 °C (Supplementary Figure 11a and b) and is less pronounced when the IMAC purified protein preparation is further purified by preparative size-exclusion chromatography (Supplementary Figure 11c and e). This suggests that during prolonged storage the purified CitAP-BsLA is most likely proteolytically cleavaged by a co-purified protease of the host used for heterologous expression. The resulting 20 kDa band can be assigned to a mixture of the CitAP-PAS domain and the BsLA domain, proteolytically cleaved in the linker region that contains a Factor Xa protease recognition site (Supplementary Figure 11a, sequence position approx. 174, sequence: IEGRE). A sample stored at 20 °C for up to 9 days still shows lipolytic activity indicating that the BsLA domain is structurally intact.

Thus, in a preparation, e.g. stored for an extended period at 20 °C, the BsLA and CitAP-PAS domain will to a large degree not be covalently linked anymore, which should abolish the signal-relay between the CitAP-PAS domain and the BsLA domain thereby abolishing the functional citrate-dependent response. To address this issue, the lipolytic activity in the presence and absence of 1 mM citrate of a sample stored for 0, 4 and 9 days at 20 °C was determined (Supplementary Figure 12). While the initial sample shows a pronounced citrate-dependent response and only one band in the corresponding SDS-PAGE analysis (see upper panel of Supplementary Figure 12), the sample stored for 4 days at 20 °C shows a defined band at a lower molecular weight (20 kDa) and correspondingly attenuated citrate-dependency. After 9 days, the band corresponding to the fusion protein (38 kDa) has nearly disappeared and a well pronounced band at around 20 kDa is visible in the corresponding SDS-PAGE analysis. The corresponding sample does not show any detectable citrate-dependency.

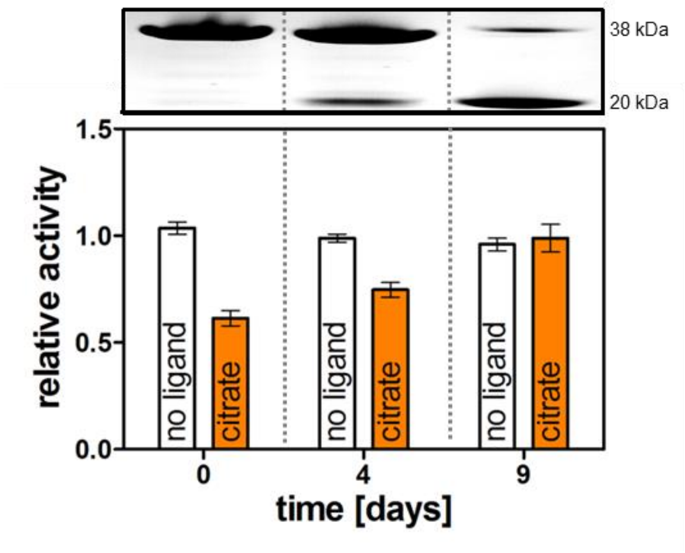

Supplementary Figure 12: Citrate-dependent activity response of an IMAC purified CitAP-BsLA sample stored for up to 9 days at 20 °C. The upper panel shows the SDS-PAGE analysis carried out using CitAP-BSLA samples stored for 0, 4 and 9 days. For clarity only the region showing the bands of the fusion protein (38 kDa) and of the proteolysis product (20 kDa) are shown. The lower panel shows the lipolytic activity of the same samples determined in either the presence (orange bars) or absence (white bars) of citrate. Lipolytic activity was measured using *p*-nitrophenylbutyrate as substrate. Error bars depict the standard deviation of the mean derived from three independent measurements.

Deconvolution of Far-UV CD spectra of CitAP-BsLA

The results of the deconvolution of CD spectra using the Convex-Constraint-Analysis Tool CCA+ [17] employing five pure secondary structural components (α-helix, random coil, parallel β-sheet, anti-parallel β-sheet and β-turn)[18,19] are presented in Supplementary Table 3. A direct comparison to the secondary structure content derived from the X-ray structures of the constituting parts (CitAP and BsLA) of the fusion protein is shown in Supplementary Table 4. The addition of Triton X100 (TX100) does not have a significant impact on the folding and secondary structure of CitAP-BsLA (Supplementary Table 3). Moreover, the secondary structural composition derived from the CD spectrum of CitAP-BsLA agrees very well with the theoretical secondary structure content derived from the X-ray structures of the components, which suggests that all structural domains of the fusion protein are well folded and functional.

Supplementary Table 3. Convex Constraint Analysis (CCA) Deconvolution of CitAP-BSLA far-UV CD data.

CitA-BSLA	α-helix	β-sheet parallel	β-sheet antiparallel	Turn and other	random coil
	% secondary structure (number of amino acids)				
- citrate	36.3 (129)	11.3 (40)	16.1 (57)	23.8 (85)	12.5 (45)
+ citrate	35.5 (126)	11.3 (40)	17.4 (62)	23.2 (83)	12.6 (45)
+ citrate + TX100	35.9 (128)	13.4 (47)	15.7 (56)	24.6 (88)	10.4 (37)
associated error (%)	0.021	0.002	0.004	0.009	0.002

Supplementary Table 4. Comparison of the CD predicted secondary structure content and the secondary structure content derived from DSSP analysis of CitAP-BSLA component structures.

	PDB ID	Number amino acids	α-helix	β-sheet	Turn, random coil and other[$]
			number of amino acids with given secondary structure		
His_6-tag[§]		20	-	-	20
DSSP CitA	2J80	129	51	47	31
Linker[&]	2J80; 2PR5	21	21	-	-
DSSP BSLA	1ISP	179	56	38	85
Sum:		349	128	85	136
CD CitA-BSLA		356	129	97	130

[$:] includes all potential other secondary structure elements such as 3-10 helices, β-hairpins etc.[§:] theoretical secondary structure of the N-terminal 20 amino acid His6-tag. [&:] The theoretical secondary structure composition was derived for the corresponding structural segments of the CitAP X-ray structure (2J80) and the corresponding YtvA X-ray structure (2PR5).

Supplementary References

1 Green, M. R., Sambrook, J. & Sambrook, J. *Molecular cloning : a laboratory manual*. 4th edn, (Cold Spring Harbor Laboratory Press, 2012).

2 Nov, Y., Fulton, A. & Jaeger, K. E. Optimal Scanning of All Single-Point Mutants of a Protein. *J Comput Biol* **20**, 990-997, doi:10.1089/cmb.2013.0026 (2013).

3 Fulton, A. *et al.* Exploring the Protein Stability Landscape: Bacillus subtilis Lipase A as a Model for Detergent Tolerance. *Chembiochem* **16**, 930-936, doi:10.1002/cbic.201402664 (2015).

4 Rahmen, N. *et al.* Exchange of single amino acids at different positions of a recombinant protein affects metabolic burden in Escherichia coli. *Microb Cell Fact* **14**, doi:ARTN 1010.1186/s12934-015-0191-y (2015).

5 Studier, F. W. Protein production by auto-induction in high-density shaking cultures. *Protein Expres Purif* **41**, 207-234, doi:10.1016/j.pep.2005.01.016 (2005).

6 Abraham, M. J. *et al.* GROMACS: High performance molecular simulations through multi-level parallelism from laptops to supercomputers. *SoftwareX* **1-2**, 19-25 (2015).

7 Li, D. W. & Bruschweiler, R. NMR-based protein potentials. *Angewandte Chemie* **49**, 6778-6780, doi:10.1002/anie.201001898 (2010).

8 Jorgensen, W. L., Chandrasekhar, J., Madura, J. D., Impey, R. W. & Klein, M. L. Comparison of Simple Potential Functions for Simulating Liquid Water. *J Chem Phys* **79**, 926-935, doi:Doi 10.1063/1.445869 (1983).

9 Wang, J., Wolf, R. M., Caldwell, J. W., Kollman, P. A. & Case, D. A. Development and testing of a general amber force field. *Journal of computational chemistry* **25**, 1157-1174, doi:10.1002/jcc.20035 (2004).

10 Sousa da Silva, A. W. & Vranken, W. F. ACPYPE - AnteChamber PYthon Parser interfacE. *BMC research notes* **5**, 367, doi:10.1186/1756-0500-5-367 (2012).

11 Darden, T., York, D. & Pedersen, L. Particle Mesh Ewald - an N.Log(N) Method for Ewald Sums in Large Systems. *J Chem Phys* **98**, 10089-10092, doi:Doi 10.1063/1.464397 (1993).

12 Feenstra, K. A., Hess, B. & Berendsen, H. J. C. Improving efficiency of large time-scale molecular dynamics simulations of hydrogen-rich systems. *Journal of computational chemistry* **20**, 786-798 (1999).

13 Eggert, T. *Die lipolytischen Enzyme LipA und LipB von Bacillus subtilis: Charakterisierung und Optimierung mit gerichteter Evolution.* , Universität Bochum, (2001).

14 van Pouderoyen, G., Eggert, T., Jaeger, K. E. & Dijkstra, B. W. The crystal structure of Bacillus subtilis lipase: a minimal alpha/beta hydrolase fold enzyme. *J Mol Biol* **309**, 215-226 (2001).

15 Petoukhov, M. V. *et al.* New developments in the ATSAS program package for small-angle scattering data analysis. *J Appl Crystallogr* **45**, 342-350, doi:10.1107/S0021889812007662 (2012).

16 Sevvana, M. *et al.* A ligand-induced switch in the periplasmic domain of sensor histidine kinase CitA. *J Mol Biol* **377**, 512-523, doi:10.1016/j.jmb.2008.01.024 (2008).

17 Perczel, A., Hollosi, M., Tusnady, G. & Fasman, G. D. Convex constraint analysis: a natural deconvolution of circular dichroism curves of proteins. *Protein engineering* **4**, 669-679 (1991).

18 Buttani, V. *et al.* Conformational analysis of the blue-light sensing protein YtvA reveals a competitive interface for LOV-LOV dimerization and interdomain interactions. *Photoch Photobio Sci* **6**, 41-49, doi:10.1039/b610375h (2007).

19 Rani, R. *et al.* Conservation of dark recovery kinetic parameters and structural features in the pseudomonadaceae "short" light, oxygen, voltage (LOV) protein family: implications for the design of LOV-based optogenetic tools. *Biochemistry-Us* **52**, 4460-4473, doi:10.1021/bi400311r (2013).
